# Historical contingency and the gradual evolution of metabolic properties in central carbon and genome-scale metabolisms

**DOI:** 10.1186/1752-0509-8-48

**Published:** 2014-04-23

**Authors:** Aditya Barve, Sayed-Rzgar Hosseini, Olivier C Martin, Andreas Wagner

**Affiliations:** 1 Institute of Evolutionary Biology and Environmental Sciences, University of Zurich, Bldg. Y27, Winterthurerstrasse 190, CH-8057 Zurich, Switzerland; 2 The Swiss Institute of Bioinformatics, Bioinformatics, Quartier Sorge, Batiment Genopode, 1015 Lausanne, Switzerland; 3 Computational Biology and Bioinformatics Master’s Program, Department of Computer Science, ETH Zurich, Universitätsstrasse. 6, CH-8092 Zurich, Switzerland; 4 INRA, UMR 0320/UMR 8120 Génétique Végétale, Univ Paris-Sud, F-91190 Gif-sur-Yvette, France; 5 The Santa Fe Institute, 1399 Hyde Park Road, Santa Fe, NM 87501, USA

**Keywords:** Genome scale metabolism, Central carbon metabolism, Genotype, Phenotype, Connectedness of genotype networks, Historical contingency

## Abstract

**Background:**

A metabolism can evolve through changes in its biochemical reactions that are caused by processes such as horizontal gene transfer and gene deletion. While such changes need to preserve an organism’s viability in its environment, they can modify other important properties, such as a metabolism’s maximal biomass synthesis rate and its robustness to genetic and environmental change. Whether such properties can be modulated in evolution depends on whether all or most viable metabolisms – those that can synthesize all essential biomass precursors – are connected in a space of all possible metabolisms. Connectedness means that any two viable metabolisms can be converted into one another through a sequence of single reaction changes that leave viability intact. If the set of viable metabolisms is disconnected and highly fragmented, then historical contingency becomes important and restricts the alteration of metabolic properties, as well as the number of novel metabolic phenotypes accessible in evolution.

**Results:**

We here computationally explore two vast spaces of possible metabolisms to ask whether viable metabolisms are connected. We find that for all but the simplest metabolisms, most viable metabolisms can be transformed into one another by single viability-preserving reaction changes. Where this is not the case, alternative essential metabolic pathways consisting of multiple reactions are responsible, but such pathways are not common.

**Conclusions:**

Metabolism is thus highly evolvable, in the sense that its properties could be fine-tuned by successively altering individual reactions. Historical contingency does not strongly restrict the origin of novel metabolic phenotypes.

## Background

For biological systems on different levels of organization, the same broadly defined phenotype can usually be formed by more than one genotype. Examples include RNA, where many genotypes (sequences) share the same secondary structure phenotype [1-4]; proteins, where multiple amino acid sequences form the same fold [5,6]; regulatory circuits, where many genetically encoded circuit topologies can form the same expression pattern [7-9]; and metabolism, where multiple metabolic genotypes, encoding different combinations of chemical reactions, can confer viability on the same spectrum of nutrients [10-13]. The number of genotypes with the same phenotype is usually astronomical. For example, it can exceed 10^20^ for moderately long RNA molecules of 40 nucleotides with the same secondary structure [14]; it has been estimated at 10^57^ for proteins that adopt a fold characteristic of the bacteriophage λ transcriptional repressor [15], and at more than 10^40^ for model regulatory circuits of 10 genes that form a given gene expression pattern [7].

The many different genotypes that share one aspect of their phenotype may differ in other aspects, such as the thermodynamic stability of a given RNA or protein fold, the resilience of a gene expression pattern to stochastic noise, or the robustness of a metabolism to deletion of genes that encode metabolic enzymes [1,7,16,17]. Because such properties can be important for the biological function of any one system, the question whether they can be “fine-tuned” in evolution is important [7,18-20]. Such fine-tuning may depend on whether one can start from any one genotype with a given phenotypic property and reach most other such genotypes through sequences of small genetic change.

Whether such fine-tuning is possible can be studied in the framework of a space of possible genotypes, where two genotypes are adjacent if they differ by the smallest possible genetic change, such as a single amino acid change in two proteins. In this framework, the question becomes whether a set of genotypes with the same phenotype forms a single connected genotype network (also known as a neutral network [1]), or whether this network fragments into multiple isolated subnetworks or *disconnected components*[21].

Whenever such fragmentation occurs, the constraint it imposes on genotypic change does not only affect the ability to modulate a phenotype. It also gives an important role to historical accidents in the evolutionary process: The genotype with a given phenotype that evolution happened to have “discovered” first can determine the number and identity of other genotypes reachable through gradual genetic change. And by restricting the number of accessible genotypes, fragmentation can also restrict the spectrum of novel phenotypes accessible as new adaptations. The reason is that this spectrum depends strongly on a genotype’s location in genotype space [22]. The further evolution can “walk away” from a given genotype, the more the spectrum of accessible phenotypes changes [1,11,23-26]. In sum, fragmentation of a genotype network can cause historical contingency and restrict a system’s potential for future evolutionary change.

Existing work, based on computational models of phenotype formation, shows that fragmentation is system-dependent. For example, in RNA secondary structure phenotypes, genotype networks are typically highly fragmented [18,27], whereas for regulatory circuits, such fragmentation depends on the kind of circuit studied, its size, and how one defines its gene expression phenotypes [7,8,28]. Because the question has thus far not been answered in metabolic systems, we here analyze the connectedness of a space of metabolisms.

A metabolism is a complex network of chemical reactions, catalyzed by enzymes and encoded by genes, whose most fundamental task is to synthesize multiple small molecule precursors for biomass, such as amino acids, nucleotides, and lipids [29,30]. An organism’s metabolic genotype is the part of a genome that encodes metabolic genes. It is thus fundamentally a string of DNA, but can be represented more compactly as a binary vector of length *N*, where *N* is the number of metabolic reactions in a known “universe” of metabolic reactions (Additional file 1[10,11], see Methods). This universe comprises all enzyme-catalyzed reactions known to take place in some organism. The *i*-th entry of this vector corresponds to the *i*-th reaction in a list of such reactions, and for any one organism, the value of this entry is one if the organism can catalyze the *i*-th reaction, and zero otherwise. On evolutionary time scales, the reaction complement of a metabolism can change through processes such as horizontal transfer of enzyme-coding genes, gene deletions, as well as gene duplications followed by sequence divergence.

The known “universe” of metabolism currently comprises more than *N* = 5000 reactions [31,32]. This means that there are more than 2^5000^ different metabolic genotypes, which constitute a vast space of possible metabolisms. For any one metabolism in this space and any one chemical environment, one can compute the spectrum of biomass precursors that it can synthesize using the constraint-based computational method of flux-balance analysis (FBA). We call any one metabolism *viable* in a specific chemical environment, if it can synthesize every single one in a spectrum of essential biomass precursors from nutrients in this environment [10,11,13,33] (see Methods). We will here consider minimal chemical environments that contain only one carbon source, such as glucose, as the sole carbon source.

Because connectedness of a metabolic genotype network may depend on the number *n* of reactions in a metabolism, we distinguish in our analysis metabolisms of different sizes. If Ω(*n*) is the set of all metabolisms with *n* biochemical reactions (*n* ≤ *N*) and if *V*(*n*) is the subset of all viable metabolisms, we are interested in whether *V*(*n*) is connected. Because the metabolisms of free-living heterotrophic metabolisms may have thousands of reactions, we need to study *V*(*n*) for metabolisms this large. This is not an easy task, because the set of viable metabolisms is so enormous that exhaustive enumeration is impossible [10,34]. Therefore, to sharpen our intuition and to illustrate key concepts, we first analyze a smaller metabolic genotype space whose viable metabolisms can be enumerated exhaustively. This is the space of metabolisms that can be formed by subsets of *N* = 51 reactions in central carbon metabolism [35] (see Methods). Even though central carbon metabolism is highly conserved, its reaction complement varies in nature, for example through variants of glycolysis [36-40] and the tricarboxylic acid cycle, where some organisms have an incomplete cycle [41]. We go beyond such naturally occurring variation and analyze metabolisms comprised of all possible subsets of all 51 reactions. Even though this number of metabolisms is astronomical (2^51^ ≈ 10^15^), we were able to determine viability for all of them, and thus analyze the connectivity of *V*(*n*) for all *n* ≤ *N* (*N* = 51). After that, we turn to larger, genome-scale metabolisms, where we study the connectivity of *V*(*n*) through a sampling approach. As many of the metabolisms used in our analysis may not be realized in extant organisms, we also refer to them as potential metabolisms.

Our observations show that for all but the simplest metabolisms, those that contain close to the minimal number of reactions necessary for viability, most viable potential metabolisms *V*(*n*) lie on a single connected genotype network. Where fragmentation into different components occurs, its biochemical cause are alternative biochemical pathways that occur in different components, that are essential for the synthesis of specific biomass precursors, that comprise more than one reaction, and that cannot be transformed into one another by changes in single reactions without destroying viability. Because such pathways only occur in the smallest potential metabolisms, fragmentation and thus historical contingency do not strongly constrain the evolution of properties such as robustness, biomass synthesis rate, or the accessibility of novel metabolic phenotypes.

## Results

### Study System 1: Central Carbon Metabolism

Our first analysis focuses on potential metabolic genotypes that can be formed with subsets of *N* = 51 reactions in the central carbon metabolism of *E. coli*[35] (see Methods). This metabolic core of *E. coli* includes reactions from glycolysis/gluconeogenesis, the tricarboxylic acid cycle, oxidative phosphorylation, pyruvate metabolism, the pentose phosphate shunt, as well as some reactions from glutamate metabolism (Additional file 2). It produces 13 precursor molecules (Additional file 2) that are required to synthesize all 63 small biomass molecules of *E. coli*, including nucleotides, amino acids, and lipids [30,35,42]. Examples of these precursors include oxaloacetate, a metabolite participating in the tricarboxylic acid cycle, which is used in the synthesis of amino acids such as asparagine, aspartate, lysine, and threonine [29,42]. Another example is ribose-5-phosphate, which participates in the pentose phosphate pathway, and is necessary for the synthesis of nucleotides and amino acids, such as histidine, phenylalanine, and tryptophan [29,42]. In our analysis, we consider a metabolism *viable* only if it can synthesize all 13 of these biomass precursors in a well-defined minimal environment containing a specific *sole* carbon source, such as glucose (see Methods).

### The fraction of viable genotypes is extremely small and decreases as metabolism size *n* decreases

For each *n* ≤ *N =* 51, we here explore the space Ω(*n*) of metabolisms (metabolic genotypes) with a given number of *n* reactions. We represent each such metabolism as a binary vector of length *N* = *51*, whose *i*-th entry is equal to one if the *i*-th reaction is present and zero otherwise. The largest metabolism (*n* = *N*) is the one where all reactions are present. The space of all possible metabolisms that contain a subset of these 51 reactions has 2^51^ (≈10^15^) member genotypes, while for a given *n*, Ω(*n*) contains $\left({}_{n}^{51}\right)$ genotypes. We are especially interested in the subset *V*(*n*) of Ω(*n*) that consists only of viable metabolisms. Because, Ω(*n*) can be very large, determining *V*(*n*) is no small undertaking. For example, for metabolisms with *n* = 30, Ω(*n*) contains more than 1.14 × 10^14^ genotypes, and the viability of each of them cannot be determined by brute force. However, one can use some peculiarities of metabolism to render this computation feasible (see Methods). For example, consider a metabolism (the “parent”) with *n* reactions and another metabolism (the “child”) derived from it by deleting one reaction. If the parent is not viable then the child will not be viable either. By analyzing the viability of metabolisms with decreasing numbers of reactions *n*, and taking advantage of this relationship, we were able to reduce the computational cost of enumerating viable metabolisms by a factor ≈ 10^6^ to the evaluation of viability for only 1.55 × 10^9^ metabolisms [43].

**Figure 1:**
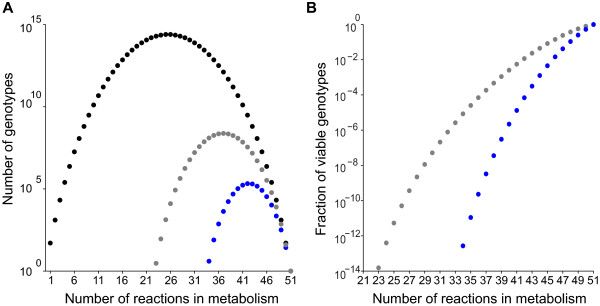
**The number of viable metabolisms *V*(*n*) decreases as the number of reactions *n *decreases. (A)** The vertical axis (note the logarithmic scale) shows the number of genotypes, and the horizontal axis shows the number *n* of reactions in a potential metabolism. Black circles represent the number of genotypes in genotype space Ω(*n*) (regardless of viability), grey circles show the number of potential metabolisms viable on glucose*,* whereas the blue circles denote the number of potential metabolisms viable on all 10 carbon sources. **(B)** The vertical axis (note the logarithmic scale) shows the fraction |*V*(*n*)| /|Ω(*n*)|. The grey circles show the fraction of genotypes viable on glucose relative to the number of possible metabolisms, whereas the blue circles denote the fraction of genotypes viable on 10 carbon sources relative to the number of possible metabolisms. Note that viable genotypes become extremely rare as the number of reactions in a metabolism decreases. Data for both figures is based on all viable metabolisms for each *n* (Additional file 3 and Additional file 8).

Figure 1A shows the number of viable metabolisms *V*(*n*) (grey circles), together with the number of all metabolisms (black circles, Ω(*n*) = $\left({}_{n}^{51}\right)$) as a function of the number *n* of reactions. Note the logarithmic vertical axis. The number of viable metabolisms has a maximum at *n* = 37 with a total of 2.39 x 10^8^ metabolisms, while the minimum size of a viable metabolism, i.e., the smallest *n* such that *V*(*n*) > 0 is 23 (Additional file 3). This means that at least 23 reactions are required to synthesize all 13 biomass precursors on glucose. There are three such smallest metabolisms, one of which is shown in Additional file 4. Figure 1B expresses *V*(*n*) as a fraction of the number of metabolisms Ω(*n*) (grey circles), and shows that this fraction decreases with decreasing *n*. This means that random sampling is much less likely to yield a viable metabolism for small than for large metabolisms. For the smallest *n* with viable metabolisms (*n* = 23), the three viable potential metabolisms correspond to a fraction 10^-14^ of all metabolisms of size 23. The largest viable metabolism contains all *n* = 51 reactions.

### Useful principles to determine the connectedness of genotype networks

The viable genotypes at any one size *n* can be represented as a genotype network, a graph whose nodes are genotypes, and where two genotypes are adjacent (connected by an edge), if they share all but one reaction. For example, the two hypothetical genotypes *G*_1_ and *G*_2_, where *G*_1_ consists of reactions {*R*_1_, *R*_2_, *R*_3_}, and *G*_2_ consists of reactions {*R*_2_, *R*_3_, *R*_4_}, are adjacent. This is because *G*_1_ and *G*_2_ share two out of the three reactions (*R*_2_ and *R*_3_). One can reach *G*_2_ from *G*_1_ by adding reaction *R*_4_ and removing *R*_1_, an event that we refer to as a reaction swap [10,12,33]. This definition of neighboring genotypes allows us to keep the number of reactions in a genotype network constant. We note that each reaction swap can be decomposed into the addition of a reaction followed by the deletion of a reaction, both of which preserve viability provided that the reaction swap does. In other words, genotype networks that are connected if adjacency is defined under reaction swaps will remain connected if adjacency is defined via a sequence of alternating reaction additions and reaction deletions.

Our principal goal is to identify whether genotype networks at any one size *n* are connected. This first requires us to establish the adjacency of $\left({}_{2}^{V\left(n\right)}\right)$ genotype pairs, followed by application of standard graph theory algorithms such as breadth-first search [21,44] to compute whether genotypes decompose into two or more disconnected components, or whether they form a single connected network, i.e., whether a path through *V(n)* exists connecting any two genotypes [21]. Because *V*(*n*) exceeds 10^6^ genotypes at intermediate *n* (Additional file 3), such conventional methods lead to large computational cost for all but the largest and smallest metabolisms (*n* = 23–28 and *n* = 46–50 reactions). For genotype networks comprising metabolisms of intermediate size (*n* = 29–45), we therefore took advantage of another relationship between “parent” and “child” metabolisms, namely that the connectivity of a genotype network at size *n* can be understood based on its connectivity at size *n*-1. We explain this relationship next.

Starting from a genotype *G*(*n*) with *n* reactions, one can obtain a parent genotype *G*(*n* + 1) with (*n* + 1) reactions by adding to it any one reaction among the *N* = 51 reactions that are not already part of *G*(*n*). Because addition of a reaction does not eliminate viability, *G*(*n* + 1) will be viable, and thus be a member of *V*(*n* + 1). For any one genotype *G*(*n*), there exist *N*-*n* reactions that are not part of this genotype. Therefore, one can obtain exactly *N-n* genotypes of size *n* + 1 by adding a single reaction to a genotype *G*(*n*). And because each pair of these genotypes of size *n* + 1 shares all but one reaction (the newly added reaction), every parent genotype in this set is adjacent to every other parent genotype. In other words, these genotypes form a clique in *V*(*n* + 1) [21].

**Figure 2:**
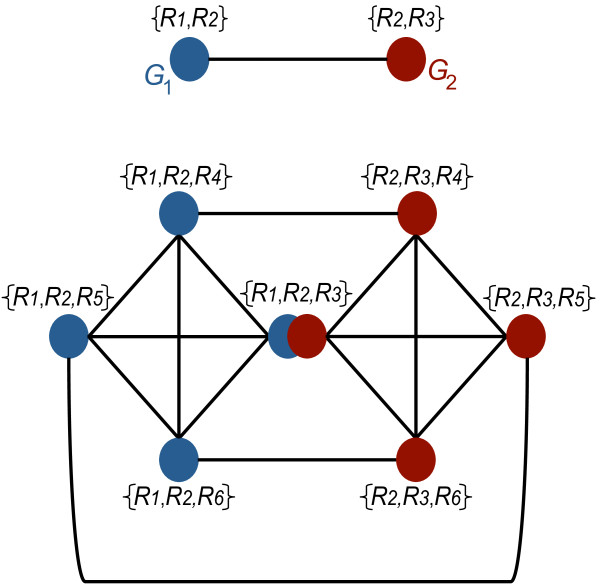
**Connectivity of potential metabolisms can be inferred from parent and child relationship.** The figure uses a hypothetical example of two neighboring metabolisms with three reactions each (upper panel) to illustrate the relationship between the connectedness of genotypes with *n* reactions (*G*(*n*)) and their “parents” of *n* + 1 reactions that can be obtained from them by adding a single reaction (lower panel). Importantly, if genotypes *G*(*n*) form a connected set, then all genotypes *G*(*n* + 1) obtained by adding one reaction to each of them also form a connected set.

We next point out that if two genotypes of size *n* are adjacent, then their corresponding genotypes of size (*n* + 1) form two cliques linked by at least one genotype of size (*n* + 1). The hypothetical example in Figure 2 illustrates this fact. Consider a “universe” of only *N* = 6 reactions - {*R*_1_, *R*_2_, *R*_3_, *R*_4_, *R*_5_, *R*_6_}. The upper half of Figure 2 shows two hypothetical genotypes (*G*_1_ in blue and *G*_2_ in red) that are viable, adjacent, and contain two reactions each (*n* = 2). Genotype *G*_1_ comprises reactions {*R*_1_, *R*_2_}, while the other genotype *G*_2_ comprises reactions {*R*_2_, *R*_3_}. The lower part of the figure shows all genotypes containing three reactions each that can be obtained from adding one reaction to genotypes *G*_1_ and *G*_2_*.* Blue genotypes are parents of *G*_1_, whereas red genotypes are parents of *G*_2_. Note that the red and blue genotypes form two cliques. Among the 7 genotypes of size *n* + 1 that are parents of either *G*_1_ or *G*_2_, one is special, because the two cliques share it. In our example, this is the genotype containing reactions {*R*_1_, *R*_2_, *R*_3_}. More generally, this shared genotype is the one genotype obtained from a pair of adjacent genotypes *G*_1_ and *G*_2_ in *V*(*n*) by adding the reaction to *G*_1_ that it does not share with *G*_2_, or vice versa. (There is only one such reaction, because *G*_1_ and *G*_2_ are adjacent). We note that additional edges connect genotypes in both cliques (Figure 2). Specifically, those edges connect the genotypes derived from adding the same reaction to *G*_1_ and *G*_2_. There are exactly (*N*-*n*-1) such edges.

These observations have the following important corollary: If a genotype network containing genotypes of size *n* is connected, then all genotypes *G*(*n* + 1) obtained from genotypes of size *n* are also connected.

So far, our line of reasoning explains connectedness of genotypes that are parents of connected genotypes at a lower size. But some viable genotypes are not parents of any other genotype. These are exactly those genotypes in which elimination of any one reaction abolishes viability. We have called such genotypes *minimal*[10,12], and note that they do not necessarily correspond to the smallest metabolisms. For example, there are 8 metabolisms that are viable on glucose and that have 24 reactions, all of which are essential (Table 1), but the smallest viable metabolisms on glucose have only 23 reactions. (We explain further below why minimal metabolisms may vary in their number of reactions.). As one increases the size of a metabolism, such “childless” metabolisms could in principle arise at any *n*. Since our preceding argument about connectedness does not apply to them, they need to be identified, and their connectedness to the rest of a genotype network needs to be examined separately, as discussed in the next section.

**Table 1:** The number of minimal metabolisms in genotype networks from the central carbon metabolism

**Number of reactions in a metabolism ( *n *)**	**Number of components**	**Number of viable metabolisms *V*** **(*n)***	**Number of minimal metabolisms**	**Fraction of minimal metabolisms**
23	2	3	3	1
24	2	91	8	0.08791
25	2	1333	23	0.01725
26	2	12512	14	0.00111
27	1	84344	27	0.00032
28	2	434238	43	9.9 x 10^-5^
29	1	1773969	28	1.57 x 10^-5^
30	1	5900578	15	2.54 x 10^-5^

The left-most column shows the number of reactions *n* in a potential metabolism, the second column from the left shows the number of disconnected components into which the genotype network of these viable metabolisms fragments, the third column shows the number of viable potential metabolisms for each *n*, and the fourth column shows the number of minimal metabolisms. Note that the fraction of minimal metabolisms (column five) decreases as metabolism size *n* increases.

We identified minimal metabolisms at each size *n* by deleting every single reaction from each genotype in *V*(*n*), and by examining whether the resulting genotype was viable, and thus identifying those genotypes in which no reaction can be deleted. Table 1 shows the number of minimal metabolisms at each *n*, and demonstrates that their proportion among all viable metabolisms (*V*(*n*)) decreases dramatically with increasing metabolism size *n*. Importantly for the next section, no minimal metabolisms viable on glucose exist above *n* = 30.

In sum, we here observed that if a genotype network is connected at size *n*, the genotype network formed by the parents of its genotypes is also connected. Because minimal metabolisms are not parents of any other metabolisms, they need to be analyzed separately.

### Metabolic genotype networks are connected for all but the smallest metabolisms

To determine connectedness of genotype networks for metabolisms *V(n)* viable on glucose, we began by analyzing the smallest (*n* = 23–28) and largest (*n* = 46–50) potential metabolisms. We did so by computing, first, edge lists for each genotype network, and, second, the connectedness of the genotype network, using the graph analysis software igraph [45]. We found that viable metabolisms of size *n* = 27, as well as *n* = 46 to *n* = 50 have only one connected component. In contrast, viable metabolisms of sizes *n* = 23, 24, 25, 26, and 28 are fragmented. They form a genotype network with two components (Table 2).

**Table 2:** Fragmentation occurs in central carbon metabolisms close to minimal number of reactions

**Number of reactions in a metabolism ( *n *)**	**Number of viable potential metabolisms *V*** **(*n)***	**Number of components**	**Fraction of viable metabolisms in the largest connected component**
23	3	2	0.667
24	91	2	0.637
25	1333	2	0.997
26	12512	2	0.992
27	84344	1	1
28	434238	2	0.977

For each metabolism size, the table shows the number of viable potential metabolisms, the number of components, and the fraction of genotypes in the largest component. The genotype network comprising metabolisms of size 23 contains three metabolisms, of which two form one component and the other is isolated from them. For metabolisms of size 24, the two components are almost of the same size, and the larger component contains 63.74 percent of the viable genotypes. For larger metabolisms, the genotype network is largely connected, with more than 97 percent of genotypes belonging to the largest component.

Fragmented genotype networks may decompose into components with different sizes, such that the majority of genotypes belong to the largest component. In this case, most viable genotypes can be reached from each other through a series of small genotypic changes that affect only single reactions each and that leave the phenotype constant. Alternatively, fragmentation of a genotype network may result in components with similar size, which can impede accessibility of many genotypes. Table 2 shows that this is not generally the case. The vertical axis denotes the fraction of genotypes belonging to the largest component of a genotype network, and it shows that the largest components of the genotype networks at size *n* = 25–28 encompass almost all (i.e., more than 99 percent) of the viable genotypes. At *n* = 23, the genotype network has two components that consist of one and two metabolisms. At size *n* = 24 there are 91 viable genotypes, 58 (64 percent) of which belong to the largest component.

**Figure 3:**
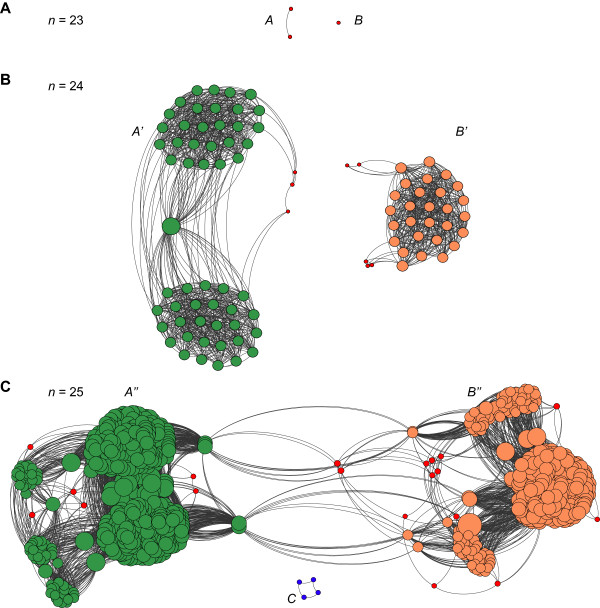
**Organization of metabolic genotype space.** The figure shows the genotype networks of potential metabolisms containing 23, 24 and 25 reactions. Each filled circle corresponds to a genotype. Two genotypes are connected by an edge (curved line) if they are neighbors. Red circles correspond to minimal metabolisms of a given number of reactions *n*. **(A)** The genotype network of size 23 is fragmented, with component *A* containing two adjacent genotypes, while component *B* contains one genotype. **(B)** Structure of the genotype network at size *n* = 24 reactions. Addition of one reaction to the two genotypes in component *A* results in genotypes of size 24 which belong to component *A’* (green), and addition of one reaction to the genotype in component *B* yields genotypes of size 24 which belong to component *B’* (orange). At size *n* = 24 reactions, eight minimal metabolisms (red circles) also arise, of which three genotypes belong to component *A’*, and five to component *B’*. Note that genotypes in components *A’* and *B’* remain disconnected. **(C)** Structure of the genotype network at size *n* = 25 reactions. Adding one reaction to all genotypes in component *A’* yields genotypes (green) in subgraph *A”*, while adding one reaction to all genotypes in component *B’* yields the genotypes (orange) of subgraph *B”*. There are 23 minimal metabolisms of size 25 (red), of which 4 genotypes form a disconnected component *C* (blue, bottom center). Note that genotypes of size 25 in subgraphs *A”* and *B”* are connected either directly or through minimal metabolisms. The size of each circle corresponds to its number of neighboring genotypes, which increases as metabolism size increases. Graphs were drawn using the graph visualization software Gephi [46].

We next turn to a more detailed analysis of genotype network fragmentation at the smallest sizes. Figure 3 shows graph representations of genotype networks whose metabolisms have sizes *n* = 23, 24 and 25. Filled circles represent genotypes. Adjacent genotypes are connected by an edge. The size of a circle corresponds to the number of neighbors of the corresponding genotype. Minimal potential metabolisms are shown in red in all three panels. All three potential metabolisms of size 23 are minimal (Figure 3A). Two of them are adjacent metabolisms and form component *A* (left), whereas the remaining isolated metabolism forms component *B* (right). The green and orange circles of Figure 3B show the result of adding one reaction from the remaining pool of 28 reactions (*N-n* = 51–23 = 28) to each of the two genotypes in component *A*. Such addition yields a connected component *A’* of 55 metabolisms with 24 reactions (green, Figure 3B). The component consists of two cliques connected to each other by a single connected genotype. Analogous addition of reactions to the single genotype in component *B* of Figure 3A yields a connected component *B’* with 28 potential metabolisms (orange) of size 24. The total number of genotypes in component *A’* and *B’* is 83. However, there are 91 viable metabolisms with size 24 (Table 1). It turns out that the missing eight metabolisms are minimal (red) and cannot be derived using reaction addition to metabolisms at size 23. Three of them are connected to component *A’* and five of them to component *B’* (Figure 3B). Overall, the number of components at size 24 reflects the number of components at size 23, because these components are derived from the smaller components at size 23. This, however, is no longer true for the genotype network of metabolisms with 25 reactions in Figure 3C. In this panel, genotypes shown in green (components *A”*) and orange (components *B”*) are parents of the green and orange genotypes in components *A’* and *B’* respectively. Notice that these components are now connected, in contrast to their disconnectedness at size 24. What connects them are some of the minimal metabolisms that arose anew at size 24 and 25. There are 23 such child-less minimal genotypes at size 25 (Figure 3C and Table 1). Four of them form a new component labeled *C* (center bottom of Figure 3C).

An analogous analysis of metabolisms up to size 30 can help understand why all larger metabolisms must be connected (Additional files 5 and 6). There are two germane observations. First, at size *n* = 30, there are approximately 5.9 × 10^6^ metabolisms and all of them fall into a single connected component (Table 1). Second, no minimal metabolisms exist at size 31 and beyond (Table 1). This means that all parent metabolisms at size (*n* + 1) are derived from child metabolisms at sizes beyond *n* = 30. By our argument in the preceding section, they must therefore form a single connected component (Figure 2).

In sum, we showed that genotype networks formed by different central carbon metabolism variants are connected in metabolic genotype space for all but the smallest viable metabolisms. With few exceptions, wherever fragmentation occurs, more than 99 percent of genotypes belong to the largest component. This high connectivity arises from the parent–child relationships we discussed, as well as from the relatively small number of minimal metabolisms that arise at each *n* (Figure 2 and Table 1).

### Essential pathways cause genotype network fragmentation

Thus far, our analysis focused on broad patterns of genotype network fragmentation. We next discuss the possible mechanistic reasons for such fragmentation. They revolve around different biochemical pathways that are essential for viability among metabolisms in different components. Essential reactions are those whose removal results in a loss of viability (see Methods), and a reaction’s essentiality may depend on other reactions present in a metabolism. That is, a reaction can be essential in one potential metabolism, but nonessential in another potential metabolism, because of the presence of alternative metabolic routes [13]. The fraction of metabolisms of a given size in which a reaction is essential is a useful quantifier of the reaction’s essentiality, which we have called the reaction’s superessentiality index [13]. The concept of (super)essentiality can be extended to entire metabolic pathways, groups of essential reactions that share substrates/products with each other and cannot be replaced without a loss of viability.

We next illustrate with an example how pathway (super) essentiality causes fragmentation of genotype networks, by demonstrating the existence of alternative essential pathways in different network components for metabolisms with 23 and 24 reactions. To identify such pathways, we first computed the superessentiality index of reactions in potential metabolisms of size 23 and 24 each, and did so for all genotypes in each of the two genotype network components (Figure 3A and B) separately (see Methods). We then examined which reactions differ in their superessentiality index between the two components. We found five such reactions, which can be subdivided into groups of two and three reactions, respectively. The first group comprises the reactions catalyzed by transketolase 1 (TKT1) and transaldolase (TALA). They are essential in all metabolisms from network component *A’* (Figure 3), but inessential in all metabolisms belonging to component *B’* . The second group comprises the reactions catalyzed by the enzymes glucose-6-phosphate dehydrogenase (G6PDH), 6-phosphogluconolactonase (PGL), and phosphogluconate dehydrogenase (GND). They are essential in all metabolisms of component *B’*, but inessential in any of the genotypes in component *A’* (Figure 3). Taken together, this means that TKT1 and TALA form a small but essential pathway in the genotypes belonging to component *A’*, while G6PDH, PGL and GND form another essential pathway in genotypes belonging to component *B’*.

**Figure 4:**
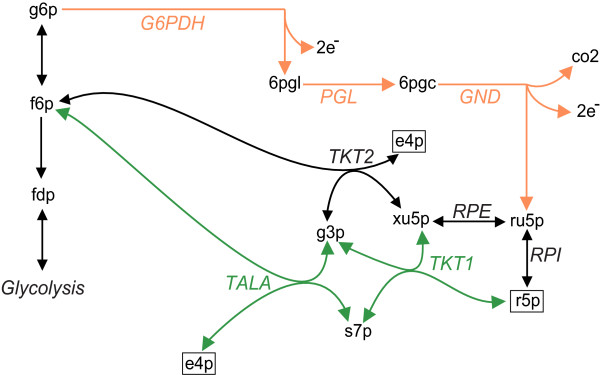
**Essential pathways in pentose phosphate metabolism.** The figure shows the two essential pathways (orange and green) in pentose phosphate metabolism that are necessary for the synthesis of biomass precursors e4p and r5p (in square boxes). Reactions in black are essential regardless of the metabolism in which they occur. Reactions catalyzed by G6PDH, PGL and GND form an essential pathway (orange), while reactions catalyzed by TALA and TKT1 (green) form another essential pathway. If the reactions in orange are absent, the reactions in green become essential and vice versa. This is because removal of TALA and TKT1 requires the synthesis of r5p through the reactions catalyzed by G6PDH, PGL and GND, while removal of these reactions forces the synthesis of r5p through TAL and TKT1. Note that metabolites g6p, f6p and g3p also participate in glycolysis and therefore can be produced there and supplied to the pentose phosphate pathway. Enzymes catalyzing each of the reactions are shown in uppercase italic typeface. Abbreviations - g6p, D-glucose-6-phosphate; r5p, D-ribose-5-phosphate; e4p, D-erythrose-4-phosphate; f6p, D-fructose-6-phosphate; fdp, fructose-diphosphate; g3p, glyceraldehyde-3-phosphate; G6PDH, glucose-6-phosphate dehydrogenase; PGL, 6-phosphogluconolactonase; GND, 6-phosphogluconate dehydrogenase; RPI, ribose-5-phosphate isomerase; RPE, ribose-5-phosphate 3-epimerase; TKT1, transketolase 1; TALA, transaldolase; TKT2, transketolase 2.

These five reactions are part of the pentose phosphate pathway, as shown in Figure 4. The pentose phosphate pathway is required for the synthesis of two biomass precursors, ribose-5-phosphate (r5p) and erythrose-4-phosphate (e4p) (solid squares in Figure 4). The reactions shown in black are essential in potential metabolisms belonging to both genotype network components (Figure 3A and B). In contrast, the essentiality of reactions participating in the two alternative essential pathways (green and orange), which contain the reactions discussed in the preceding paragraph, depends on which of the two components a potential metabolism belongs to. To understand why, we first note that the metabolites glucose-6-phosphate (g6p), fructose-6-phosphate (f6p), and glyceraldehyde-3-phosphate (g3p) are also synthesized by reactions in glycolysis, and thus constitute metabolic inputs to the pentose phosphate pathway for the synthesis of e4p and r5p. Flux balance analysis can be used to show that reactions catalyzed by transketolase 1 (TKT1) and transaldolase (TALA) are required to synthesize sufficient r5p for viability (Table S1 - biomass reaction) upon removal of any one reaction from the orange pathway (G6PDH, PGL, GND), thus rendering the reactions catalyzed by TKT1 and TALA essential. Conversely, removal of any one reaction from the green pathway (TKT1, TALA) leads to a requirement for all reactions in the orange pathway to produce the pathway output. In sum, the genotypes of size 23 and 24 are disconnected because alternative essential pathways exist in them that consist of more than one essential reaction, and because no one reaction in one pathway can replace a reaction in the other pathway. Put differently, loss of any one reaction in one pathway can only be compensated by addition of all reactions of the other pathway.

Because metabolisms at size 23 are separated by three swaps, genotype space can be connected at size 25 (subgraphs *A”* and *B”*), that is, after successive addition of two reactions.

In Additional files 5 and 7 (section - Essential pathways cause genotype network fragmentation) we discuss another example, which illustrates that essential and alternative metabolic routes need not contribute to biosynthesis of the same precursors, and may arise in functionally different and unrelated parts of metabolism. These differences notwithstanding, the examples illustrate the mechanistic reason for genotype network fragmentation: It is not possible to interconvert two genotypes in different components by one reaction swap because such interconversion will inevitably create unviable genotypes in which two alternative essential pathways are incomplete.

As a corollary, the longer such essential alternative pathways are, the greater the number of reactions *m* that need to be added to non-adjacent viable genotypes *G*(*n*), such that viable genotypes *G*(*n* + *m*) become connected.

### Metabolisms viable on multiple carbon sources are also mostly connected

Many organisms are viable on multiple carbon sources, which may impose additional constraints on a metabolism. We wished to find out how severely these constraints affect genotype network connectivity in our analysis of central carbon metabolism. To this end, we analyzed metabolisms that are a subset of our *N* = 51 reactions and that are viable on a total of 10 common carbon sources when each of them is provided as the sole carbon source (see Methods for all these carbon sources). Because glucose is among these 10 carbon sources, metabolisms viable on all 10 carbon sources are also viable on glucose. In other words, the genotype network they form at any specific metabolism size *n* is a subset of the genotype network of metabolisms viable on glucose. We note that the metabolism comprising all *N* = 51 reactions is viable on all 10 different carbon sources.

We used an approach identical to that described above for glucose to identify potential metabolisms viable on all 10 carbon sources. Their numbers are shown in Figure 1A (blue circles), which shows that, first, no metabolism with fewer than *n* = 34 reactions is viable on all 10 carbon sources, whereas the minimal size is much smaller (*n* = 23) for metabolisms viable on glucose alone (Additional file 3, Additional file 8, Table 1). Second, the number of metabolisms viable on 10 carbon sources is much smaller than the number of metabolisms viable on glucose. It reaches a maximum at *n* = 42 with 2.1 × 10^5^ metabolisms (Additional file 8), many fewer than for viability on glucose (2.39 × 10^8^ metabolisms at *n* = 37). This difference is also highlighted in Figure 1B whose vertical axis represents genotypes viable on glucose (grey) and on all ten different carbon sources (blue) as a fraction of all genotypes. At the minimal size of *n* = 34 reactions, genotypes viable on all 10 different carbon sources comprise approximately one 10^-8th^ of those genotypes viable on glucose of the same size.

This strong constraint on metabolisms viable on multiple carbon sources raises the possibility of genotype network fragmentation. However, we found no evidence for such fragmentation. Because the number of genotypes viable on 10 carbon sources is relatively small, we were able to use standard algorithms to determine their connectedness, which show that genotype networks of all sizes except for *n* = 35 and 36 reactions consist of only one connected component. At size *n* = 35 the genotype network fragments into three components. The largest of them contains 91.13 percent of viable genotypes. At size *n* = 36, the network fragments into two components, with the larger containing 99.6 percent of genotypes. This implies that one can access any metabolic genotype viable on 10 carbon sources, regardless of its size, from most other viable genotypes through a series of individual reaction changes.

### Study system 2: Genome-scale metabolisms

We have thus far studied connectedness for potential metabolisms drawn from the reduced reaction set of central carbon metabolism, which comprises a small subset of the more than 1000 reactions in the typical metabolism of a free-living organism. In this section, we focus on the connectedness of larger, genome-scale potential metabolisms. Their reactions come from the known “universe” of possible biochemical reactions, which comprises, at our present state of partial knowledge, already more than 5000 reactions [31,32]. For any one such metabolism to be viable, we require that it is able to synthesize all 63 essential biomass precursors of *E. coli*[30] – most of which are molecules central to all life, such as nucleotides and amino acids (see Methods) – in a minimal environment containing glucose as the sole carbon source.

Using our binary representation of a metabolic genotype (Additional file 1), the number of possible genome-scale metabolisms is greater than 2^5000^, which renders exhaustive analysis of connectivity infeasible. Random sampling of the space using Markov Chain Monte Carlo (MCMC) methods can be very useful [10,11,13,33], but it is not suitable for our purpose, because the MCMC approach samples genotypes from the same component of a genotype network.

**Figure 5:**
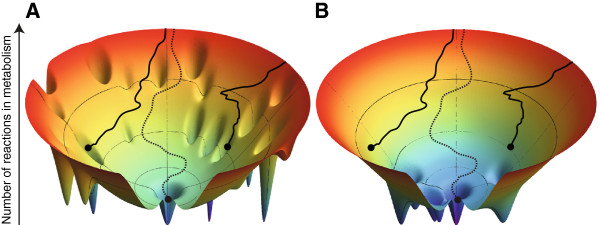
**A spatial schematic of genotype network connectivity at different metabolism sizes *n*.** Each panel shows a funnel-like landscape, where the funnel’s width reflects the number of potential metabolisms that are viable at any given number *n* of reactions (altitude in the landscape). The lowest points of the landscape correspond to potential metabolisms whose size are close to the smallest possible *n* needed for viability. Solid and dotted lines denote random sequences of reaction removal from some viable starting metabolism (not shown) that terminate at a particular size *n* and result in some viable metabolism denoted by circles. Depressions in the funnel correspond to disconnected components of the genotype network. Disconnected components in **(A)** arise at higher metabolism sizes than in **(B)**. Two of the trajectories in A (solid lines) terminate in such depressions, that is, in potential metabolisms that are part of a disconnected component. These metabolisms are not interconvertible through viability preserving reaction swaps. The same trajectories in B terminate at a part of the funnel where all viable metabolisms are still connected. The figure was generated using a script from (http://www.oaslab.com/Drawing_funnels.html)

We thus use a different sampling approach [10,12,33,47], which starts from a “global” metabolism that comprises all reactions in the known universe (and is viable on glucose). This metabolism has 5906 reactions. Its viable children would form a single connected component, but as one reduces their number of reactions further, the set of viable genotypes *V(n)* might become disconnected. Figure 5A illustrates this possibility schematically. It shows a funnel-like landscape whose width at a given number of reactions *n* (vertical axis) indicates the number of viable metabolisms at this *n*. The number of viable metabolisms approaches zero as *n* approaches the smallest possible size at which a metabolism can be viable. Starting from the global metabolism, one can randomly select a sequence of reactions for deletion while requiring that each deletion retain viability. Parts of three hypothetical deletion sequences are shown as three trajectories in the panel. Two of them (solid) lead into deep depressions in the funnel, which correspond to disconnected components of a genotype network. More precisely, a metabolism that resides in one such depression cannot be converted into another viable metabolism without changing its number of reactions (the altitude in the landscape), as doing so would require it to traverse the exterior of the funnel. The third trajectory (dotted line) enters such a depression only at a much lower number of reactions. We wanted to know whether such funnels appear in the landscape at moderate *n* (Figure 5A) or only at values of *n* close to the smallest number of reactions permitting viability (Figure 5B).

To find out, we derived multiple viable metabolisms with a given size *n* as follows. Starting from the global metabolism, we repeatedly deleted randomly chosen reactions from it, such that each deletion preserved viability, until we had arrived at a minimum metabolism, that is, a metabolism whose number of reactions cannot be reduced further. In doing so, we kept track of the deleted reactions, and the sequence in which they were deleted. Each minimum metabolism created in this way had fewer than 400 reactions (see also below). We used these minimal metabolisms, as well as information about the sequence in which reactions were deleted, to create larger potential metabolisms of varying sizes *n*, each of which corresponds to a specific point in the deletion sequence. We repeated this procedure 500 times, which allowed us to create 500 minimal metabolisms, as well as 500 potential metabolisms of various intermediate sizes.

### Most viable genome-scale metabolisms reside in the same connected component

If genotype networks were highly fragmented at a given size *n*, then different random deletion sequences would yield potential metabolisms that reside in different components of a genotype network. In this case, it would not be possible to connect two metabolisms that reside in different components of a genotype network through a sequence of reaction swaps, each of which preserves viability. With these observations in mind, we attempted to connect metabolisms in our samples of viable metabolisms of a given size (see Methods). Specifically, for any sample of metabolisms [*G*_1_, *G*_2_, *G*_3_, … , *G*_500_], we attempted to connect *G*_i_ and *G*_i+1_ (1 ≤ i < 500) through viability-preserving reaction swaps. We did this for 500 potential metabolisms of size 1400 (similar to that of *E. coli*), 1000, 500, and 400 (above the size of minimal metabolisms, see below). In this way, we were able to show that all 500 potential metabolisms are connected at each of these sizes. Thus, down to a size of *n* = 400 reactions, the genotype network of metabolisms viable on glucose is not highly fragmented, and one component comprises the vast majority or all metabolisms.

Because many free-living microorganisms are viable on multiple carbon sources, we generated 500 additional potential metabolisms through the reaction deletion process just described, but with the additional constraint that they remain viable on ten sole carbon sources (the same ten as used in our analysis of central carbon metabolism). Specifically, we created again potential metabolisms of size 1400, 1000, 500, 450 and 425 (slightly above the size of minimal metabolisms for viability on 10 carbon sources). We then repeated the procedure that attempts to connect genotypes *G*_i_ and *G*_i+1_ through viability-preserving reaction swaps. In this way, we were able to show that all 500 potential metabolisms are connected at each of these sizes. Thus, down to a size of *n* = 425 reactions, the genotype network of most metabolisms viable on all 10 carbon sources consists of one connected component.

It is possible to make this point more quantitatively and establish a statistical bound on the fraction of potential metabolisms contained in the largest connected component of *V(n)*. Specifically, let us consider the null hypothesis that more than one percent of *V(n)* resides outside this largest component. If this null hypothesis is correct, then the probability *p* that a randomly drawn viable genotype is *not* on this largest component is greater then *p* = 0.01. Moreover, the probability that some number *M* of genotypes drawn at random from *V(n)* all fall on the largest connected component would be smaller than (1-*p*)^*M*^. In our case, *M* = 500 and (1-*p*)^*M*^ < 0.99^500^ = 0.0066. In other words, the results of our sampling allow us to reject the above null hypothesis at a significance level smaller than 1 percent.

### Minimal metabolism size can help explain connectedness

**Figure 6:**
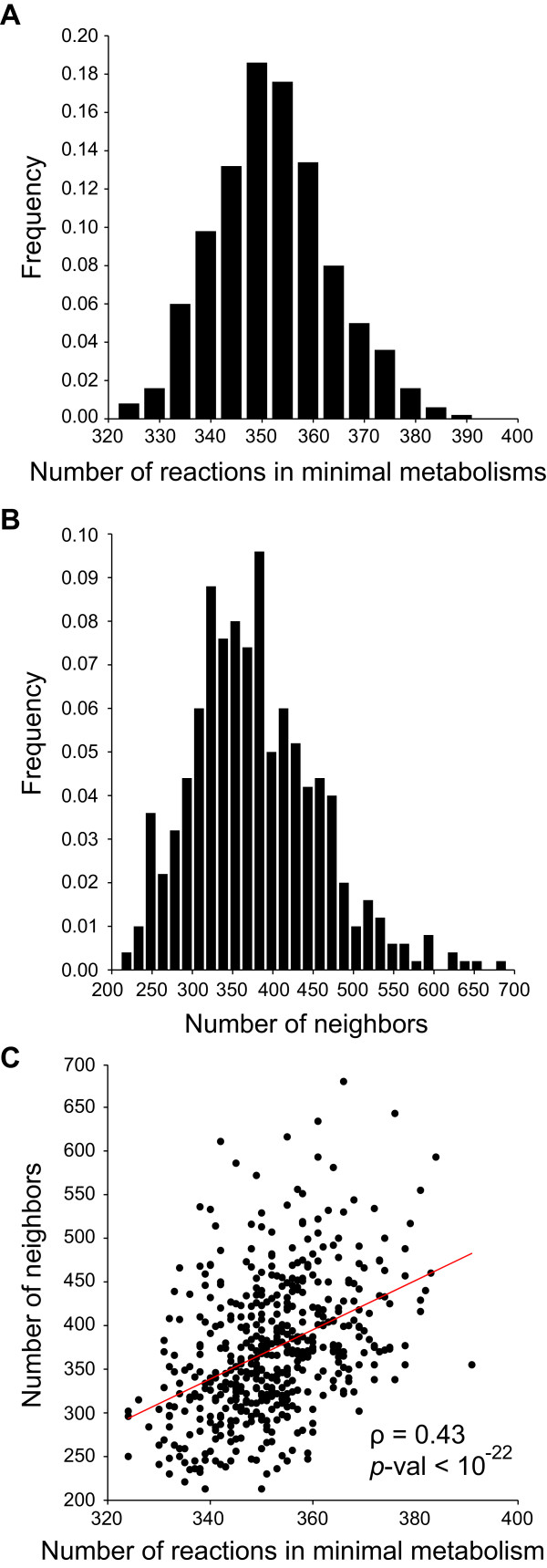
**Minimal metabolisms from the complete universe can have many viable neighbors. (A)** The horizontal axis denotes the size of minimal metabolisms and the vertical axis denotes their frequency. The average minimal metabolism comprises 352 reactions, while the largest minimal metabolism we find has 391 reactions. **(B)** The vertical axis shows the frequency of potential metabolisms with a given number of neighbors (horizontal axis). A minimal metabolism has 372.8 viable neighbors on average. Data in **(C)** show that the number of viable neighbors (vertical axis) is positively correlated with the number of reactions present in a minimal metabolism (horizontal axis). Data in **(A)**, **(B)** and **(C)** are based on 500 minimal metabolisms generated through the random reaction deletion process described in the text.

In the sections on central carbon metabolism we showed that new components disconnected from the remainder of a genotype network can arise as one increases metabolism size, and that they originate from “childless” minimal metabolisms which appear at a given size *n* that is small compared to the total number of possible reactions. To examine their size for larger metabolic system, we studied the 500 minimal metabolisms that we derived from the sequential random deletion strategy described in the previous section (Figure 6A). Their size ranges from 324 to 391 reactions, with a mean of 352 reactions (standard error = 11.44 reactions) (Figure 6A). Although we cannot absolutely exclude the possibility that minimal metabolisms exist with more than 400 reactions, the fact that all of the minimal metabolisms we found have fewer reactions suggests that the emergence of new genotype network components will be rare above 400 reactions. This observation further supports our assertion that most metabolisms with more than 400 reactions will be part of a single genotype network. It also means that essential alternative metabolic pathways of more than one reaction that are characteristic for a given connected component exist only for small metabolisms. Alternative pathways for the synthesis of most biomass molecules undoubtedly exist, but most of them can be converted into one another through sequences of single reaction changes that preserve viability.

In a final analysis, we asked whether the minimal networks that our approach identified are isolated in metabolic genotype space Ω(*n*), or whether they might themselves form large components. To this end, we simply asked whether these networks have any viable neighbors in Ω(*n*), metabolisms that differ by a single reaction swap, which are also viable. The result (Figure 6B) shows that even minimal metabolisms have typically hundreds of neighbors. Specifically, an average minimal metabolism has 372.8 viable neighbors (standard deviation: 79 neighbors). The maximum number of neighbors for a minimal metabolism is 685. Figure 6C shows that larger minimal metabolisms tend to have more neighbors than smaller ones (Spearman’s ρ = 0.43, *p*-value < 10^-22^). Taken together, this means that minimal metabolisms themselves must form large components and are certainly not isolated. It mirrors the situation in central carbon metabolism, where newly emerging minimal metabolisms at a given size *n* also form connected components, albeit small ones (Figure 3).

## Discussion

To our knowledge, our analysis of all possible ≈ 10^15^ metabolisms comprising subsets of reactions in central carbon metabolism is the first exhaustive analysis of a metabolic space this large, even though smaller-scale analyses were carried out before with different goals [48-50]. Our analysis focused on metabolisms viable on glucose, which are required to synthesize 13 products of central carbon metabolism that are biomass precursors. We found that viable metabolisms could have fewer than half (23) of the maximal number of 51 reactions in central carbon metabolism. Moreover, for metabolisms covering 77 percent of the size of the viable range (*n* = 23–51), all (*n* = 29–51) or the vast majority of metabolisms of size *n* form a single connected component (network) in the space of metabolisms.

In genome-scale metabolisms, where exhaustive enumeration is no longer possible, and where we required the synthesis of 63 common biomass molecules for viability, we found viable potential metabolisms with as few as 324 reactions, and for 93.23 percent of the size range of viable metabolisms (*n* = 400–5906) the vast majority of potential metabolisms form a single connected component of a genotype network. More specifically, with a probability of greater than 0.99, more than 99 percent of all viable metabolisms exceeding 400 reactions are part of the same component. We note that it would have been sufficient to perform the sequential reaction deletion procedure needed to arrive at this conclusion for metabolisms of size *n* = 400, and not also for metabolisms of size *n* = 400–1400, as we did. The reason is an elementary observation we made about metabolic genotype space: If a set of viable metabolisms *V*(*n*) is connected for some number of reactions *n*, then *V*(*m*) must be connected for all *m* > *n*, provided that no new minimal metabolisms appear at any value of *m*. The largest minimal metabolism we found has *n* = 391 reactions, and while we cannot exclude the existence of minimal metabolisms above *n* = 400 with certainty, such potential metabolisms would be increasingly rare at large *n*. They would create new genotype network components that would comprise a vanishing fraction of the rest of the connected genotype network (even though they might contain many potential metabolisms in absolute numbers).

Figure 5B illustrates schematically the dependence of fragmentation on metabolism size *n* that we observed. Depressions in the funnel-like landscape whose width reflects the number of viable potential metabolisms correspond to disconnected metabolic networks and appear only at small altitudes (metabolism sizes). That is, the hypothetical landscape of Figure 5B reflects our observations, whereas that of Figure 5A, where disconnected metabolisms appear at much higher reaction numbers does not.

 While we study only viability on a carbon source, other metabolic properties such as mutational robustness and access to novel phenotypes are also important [10,11] and may differ in different components. In such cases, historical contingency may indeed play a role towards the fine-tuning of metabolic properties and would be relevant in a scenario depicted by Figure 5A. However, as fragmentation occurs only at lower metabolism sizes, historical contingency may not constrain the overall evolution of metabolic systems sharply.

With possible exceptions in some marine bacteria [51,52] metabolisms with sizes as small as *n* = 400 are not usually found in free-living organisms. They occur in (endo)symbionts [53,54] and (endo)parasites [55,56], which live in close association with a host organism and are provided nutrients and a constant environment which allows them to shed many enzyme-coding genes [47,57-60]. Organisms that have lived inside a host for a long time experience less of the kinds of evolutionary change – especially horizontal gene transfer – that is powerful in endowing the genomes of free-living organisms with new evolutionary adaptations [57,59]. In other words, the fragmentation of genotype networks that we see for very small potential metabolisms, and that can constrain their evolution, is of little relevance for the evolution of free-living organisms. Those organisms whose evolution it could constrain the most are already subject to little evolutionary change for ecological reasons.

Our analysis of genotype network fragmentation provides a coarse, statistical view on the organization of genotype space. This view needs to be complemented by a mechanistic perspective that asks what distinguishes the metabolisms that exist in different components of a genotype network? What could prevent evolution from converting them into each other through a series of single viability-preserving reaction changes? The answer lies in alternative metabolic pathways that are essential for the biosynthesis of one or more biomass molecules. Potential metabolisms in one genotype network component have one such pathway, and potential metabolisms in the other component have another such pathway. (In addition, these potential metabolisms may differ in other essential pathways.) At least one of these pathways must comprise more than one reaction, otherwise the two metabolisms could be converted into one another through a single reaction swap. We have provided two examples, one involving the biosynthesis of erythrose-4-phosphate and ribose-5-phosphate through variants of the pentose phosphate pathway, the other concerning the biosynthesis of phosphoenolpyruvate.

For two reasons, such alternative essential pathways are not likely to hamper the evolution of most metabolic systems. First, we observed fragmentation only for relatively small metabolisms, which means that in larger metabolisms, alternative *essential* pathways with more than one reaction do not exist. They can usually be converted into each other by single reaction changes that do not cause a loss of viability. Second, our analysis required that we impose change through reaction swaps – a reaction addition paired with a deletion – that leave reaction numbers constant. However, this is not usually how evolutionary change in a metabolism’s reactions occurs. For example, horizontal gene transfer frequently adds more than one gene and thus more than one reaction to a metabolism [61-63]. In a metabolism that harbors one of two alternatives for an essential pathway, a horizontal gene transfer event may introduce the genes of the other pathway. After that, the two pathways may coexist, and the first pathway is free to deteriorate through loss of function mutations in its genes. A potential example of co-existing alternative pathways involves the two pathways responsible for synthesizing isopentenyl diphosphate (the 1-deoxy-D-xylulose 5-phosphate pathway and the mevalonate pathway), a molecule that is required for the synthesis of isoprenoids. Some actinomycetes that harbor both pathways in their complete forms may have obtained the responsible genes through horizontal gene transfer [64]. In sum, common forms of genetic change can help bridge different components of a genotype network where such components exist.

The main limitation of our work comes from the enormous computational cost associated with evaluating the viability of many metabolisms. While our sampling approach for genome-scale metabolisms allowed us to circumvent this problem for any one carbon source, it is possible that viability on a broader range of carbon sources (or sources of other chemical elements) might have led to greater genotype network fragmentation. This possibility is suggested by our analysis of central carbon metabolism, where metabolisms viable on 10 carbon sources must have at least 34 reactions, and fragmentation of genotype networks stops at 37 reactions. However, for genome-scale metabolisms, the metabolism sizes at which fragmentation would cease would increase only modestly with each additional carbon source on which viability is required. This is because previous work has shown that viability on every additional carbon source requires on average the addition of only two reactions to a metabolism [65]. For example, viability on ten additional carbon sources would increase the size of minimal metabolisms by only 20 reactions. Because the number of minimal metabolisms that arise *de novo* with increasing metabolic complexity *n* is closely linked to the metabolism size at which fragmentation occurs, viable genotype networks *V*(*n*) would still remain connected over the vast majority of the range of *n*. Indeed, we found that genome-scale metabolisms viable on 10 carbon sources and comprising 425 reactions are connected in genotype space and belong to the same component. Possible exceptions might involve metabolisms viable on hundreds of different carbon sources, but even environmental generalists are typically not viable on that many. (The generalist *E. coli* is viable on some 50 alternative carbon sources [30]).

Another limitation of our work is that we only considered viability on carbon sources. We cannot exclude the possibility that viability on sources of different chemical elements may lead to different fragmentation patterns. However, it is unlikely that carbon sources are exceptional in this regard. For example, the minimal size of metabolisms viable on different sulfur sources comprises only 90 reactions, and is thus even smaller than that of metabolisms viable on carbon [12]. The reason is that fewer biomass molecules contain sulfur, an observation that also holds for the two other key elements nitrogen and phosphorus.

A further limitation is that we focus on evolutionary constraints caused by the presence or absence of biochemical reactions, rather than on differences in the regulation of existing enzymes or their encoding genes. Such regulatory constraints can influence important metabolic properties such as biomass growth rate [20]. However, they can also be easily broken through regulatory evolution, even on the short time scales of laboratory evolution experiments [19,20,66]. Reaction absence is thus a more fundamental constraint, but we note that the exploration of regulatory constraints remains an important task for future work. Moreover, to understand connectedness as a function of reaction numbers, we had to preserve reaction numbers and analyze connectedness through reaction swaps. We note that a reaction swap can be considered as an addition of a reaction, which does not change viability and a reaction deletion that preserves viability. That is, every reaction swap can be broken down into two biologically relevant changes, and thus genotype network connectivity resulting from reaction swaps also holds for single reaction additions and deletions.

Finally, we do not consider one potential cause of genotype network fragmentation: If one required for viability that biomass precursors need to be synthesized at a high rate, then genotype networks may fragment more often than we observe. However, fast biomass synthesis and its main consequence, rapid cell division, are not universally important outside the laboratory environment. For example, a survey of microbial growth rates shows that many microbes have very long generation times in the wild [67]. Rapid growth thus may not be a biological sensible requirement for viability in many wild organisms.

## Conclusions

In sum, our analysis has shown that over a broad range of metabolic complexity, historical contingency is not likely to strongly constrain the modulation of metabolic properties, or the accessibility of novel metabolic phenotypes. Only the smallest metabolisms, which typically do not occur in free-living organisms, are likely to be subject to such constraints, which stem from genotype network fragmentation. Additional factors that we did not consider explicitly are likely to further reduce such fragmentation. They include pervasive promiscuous enzymes, which are capable of catalyzing more than one biochemical reaction [68,69], and horizontal gene transfer events that can add multi-reaction metabolic pathways to an existing metabolism, and thus bridge otherwise disconnected genotype network components.

## Methods

### Flux balance analysis

Flux balance analysis (FBA) is a constraint-based computational method [34,70] that can predict synthetic abilities and other properties of metabolisms – complex networks of enzyme-catalyzed biochemical reactions. Any one such network can comprise anything from a few dozen reactions, such as central carbon metabolism [70], to the thousands of reactions in a complex genome-scale metabolism. FBA uses information about the stoichiometry of each reaction to predict steady state fluxes for all reactions in a metabolic network. The necessary stoichiometric information is represented as a stoichiometric matrix, *S*, of dimensions *m × n*, where *m* denotes the number of metabolites, and *n* denotes the number of reactions in a metabolism [34,70]. FBA assumes that the concentrations of intracellular metabolites are in a steady state, which allows one to impose the constraint of mass conservation on them. This constraint can be written as *Sv* = 0, where *v* denotes a vector of metabolic fluxes through each reaction in a metabolism. The above equation has a large space of possible solutions, but not all of these solutions may be of biological interest. To restrict this space to fluxes of interest, FBA uses linear programming to maximize a biologically relevant quantity in the form of a linear objective function *Z*[70]. Specifically, the linear programming formulation of an FBA problem can be expressed as

$$\max\;Z=\max\left\{c^{T}v|\mathrm{Sv}=0,a\;\leq \;v\;\leq \;b\right\}$$

The vector *c* contains the set of scalar coefficients that represent the maximization criterion. The individual entries of vectors *a* and *b*, respectively, contain the minimal and maximally possible fluxes for each reaction in *v*. Irreversible reactions can only have fluxes with positive signs, whereas irreversible reactions can have fluxes of both signs.

We are here interested in predicting whether a metabolism can sustain life in a given spectrum of environments, that is, whether it can synthesize all necessary small biomass molecules (biomass precursors) required for survival and growth. For our analysis of central carbon metabolism, there are 13 such essential precursors (Additional file 2 and [35]). For our analysis of genome scale metabolisms, we use all 63 [30] biomass precursors of *E. coli*, because most of them would be required in any free-living organism. They include 20 proteinaceous amino acids, DNA and RNA nucleotide precursors, lipids and cofactors. We use these biomass precursors to define the objective function and the vector *c*. We employed the package CLP (1.4, Coin-OR; https://projects.coin-or.org/Clp) to solve linear programming problems. The computer program required for FBA is available in Additional file 9.

### Growth environments

Along with the biomass composition and stoichiometric information about a metabolic network, computational predictions of viability require information about the chemical environments that contain the nutrients needed to synthesize biomass precursors. In our analysis of central carbon metabolism, we consider a minimal aerobic growth environment composed of a sole carbon source, along with ammonium as a nitrogen source, inorganic phosphate as a source of phosphorus, as well as oxygen, protons, and water. When studying the viability of metabolisms on different carbon sources, we vary the carbon source while keeping all the other nutrients constant. When we say a particular metabolism is viable on 10 carbon sources, we mean that it can synthesize all biomass precursors when each of these carbon sources is provided as the sole carbon source in a minimal medium. The ten carbon sources we consider are D-glucose, acetate, pyruvate, D-lactate, D-fructose, alpha-ketoglutarate, fumarate, malate, succinate and glutamate.

Our analysis of genome-scale metabolisms requires a minimal environment with more nutrients, i.e., a sole carbon source, ammonium, inorganic phosphate, sulphate, sodium, potassium, cobalt, iron (Fe2+ and Fe3+), protons, water, molybdate, copper, calcium, chloride, magnesium, manganese and zinc [30]. For our analysis of genome-scale metabolisms viable on 10 carbon sources, we used the 10 carbon sources from the preceding paragraph.

### The reactions used in the analysis of central carbon metabolism

We use a global set of reactions in central carbon metabolism, which is based on a published reconstruction of *E. coli* central carbon metabolism [35]. From the published reconstruction [35], we deleted four reactions involved in ethanol synthesis, metabolism and transport. We also grouped the reactions catalyzed by aconitase A and aconitase B into one reaction. We did this mainly to reduce the size of the set of reactions, in order to render the exploration of all variant metabolisms derived from it feasible. The final reaction set consists of *N* = 51 intracellular reactions, and we analyzed the viability of metabolisms comprising all possible 2^51^ subsets of this set. The reconstruction in [35] also involves 20 transport reactions, which are necessary to import nutrients or excrete waste products, and which we assume to be present in all metabolisms we studied.

### The known reaction “universe” and the global metabolism

We refer to the known universe of biochemical reactions as the set of reactions known to occur in some organism based on currently available biochemical knowledge. To arrive at this set, we curated data from the LIGAND database [31,32] of the Kyoto Encyclopedia of Genes and Genomes [71], which is divided into two smaller databases, the REACTION database and the COMPOUND database. These two databases together provide information about metabolic reactions, participating chemical compounds, and associated stoichiometric information. As described previously [10,11,13,33], we curated reactions from these databases by excluding reactions involving polymer metabolites of unspecified numbers of monomers; general polymerization reactions with uncertain stoichiometry; reactions involving glycans, owing to their complex structure; reactions with unbalanced stoichiometry; and reactions involving complex metabolites without detailed structural information [71]. After curation of these reactions, we added to them all non-redundant reactions from the published *E. coli* metabolic model (*i*AF1260), which comprises 1,397 non-transport reactions [30]. At the end of this procedure, we had arrived at a set of 5,906 non-transport reactions and 5,030 metabolites. We converted this set into what we call a global metabolism by including all *E. coli* transport reactions in this set [30]. Unsurprisingly, the global metabolism can synthesize all biomass precursors of *E. coli* from any of the carbon sources we consider here. We note that this metabolism may not be biologically realizable, for example, because it may contain thermodynamically infeasible pathways. However, we merely use it as a starting point to create smaller and ultimately minimal metabolisms through the sequential reaction deletion process described below.

### Genotypes, phenotypes and viability

The genes encoding the enzymes that catalyze a metabolism’s reactions constitute the metabolic genotype of an organism. For our purpose, a more compact representation of a metabolic genotype is useful, which represents this genotype as a binary vector whose *i*-th entry corresponds to the *i*-th reaction in some global set or universe of biochemical reactions. This entry will be equal to one if an organism’s genome encodes an enzyme capable of catalyzing this reaction, and zero otherwise (Additional file 1). The genotype space of all possible metabolisms comprises 2^*N*^ metabolisms, where *N* is the total number of known or considered chemical reactions (*N* = 51 for our analysis of central carbon metabolism, and *N* = 5906 for our analysis of genome-scale metabolisms). Any one organism's metabolic genotype can be thought of as a point in this space. Genotypes (metabolisms) viable in a given chemical environment are those that can synthesize all biomass precursors from nutrients in this environment. Specifically, a metabolism is considered viable on a carbon source if its biomass synthesis rate is more than one percent of the biomass synthesis rate of the central carbon metabolism (*N* = 51) on that carbon source. We note that many of the metabolisms we study here, may not be realized in extant organisms. We thus refer to these metabolisms as potential metabolisms.

### Essential and nonessential reactions

We define a reaction as essential for viability if its elimination abolishes viability in a given chemical environment. To identify all such essential reactions in a given metabolism, we eliminated each reaction and used FBA to assess whether non-zero biomass growth flux was still achievable. For our analysis of viability on 10 different (sole) carbon sources, we defined a reaction as essential if its elimination abolishes viability on *at least one* of the 10 carbon sources. The computer program required for computing essential and non-essential reactions is available in Additional file 9.

### Identification of viable central carbon metabolisms

To identify all viable metabolisms by exhaustive enumeration of viability for all 2^51^ (10^15^) possible metabolisms in central carbon metabolism would be infeasible. Fortunately, such brute-force enumeration is also not necessary, for two reasons. The first originates from the notion of “environment-general superessential reactions” [13]. These are reactions whose elimination abolishes viability in each of the 10 carbon sources used here. To find such reactions, we converted the universe of central carbon metabolism into a format amenable to FBA analysis, as described earlier in this section. We then deleted each reaction and determined viability on each of the 10 carbon sources. We found six reactions (Additional file 2, in red) that were necessary for biomass synthesis on each source. Any viable central carbon metabolism would require all six reactions, which reduces the number of metabolisms whose viability needs to be evaluated from 2^51^ to 2^(51–6)^ = 2^45^(≈10^13^).

The second reason derives from a simple observation that reduces the number of genotypes whose viability needs to be determined even more dramatically: Removal of a reaction from an unviable metabolism cannot result in a viable metabolism. This means that among all metabolisms with *n*-1 reactions, we need to evaluate only the viability of those that are derived from viable potential metabolisms with *n* reactions through removal of one reaction. We incorporated this idea into an algorithm that allowed us to enumerate all viable genotypes [43].

### Sampling of viable genome-scale metabolisms

To sample large (genome-scale) metabolisms, we started from the global metabolism of 5,906 reactions and deleted (eliminated) from it a sequence of randomly chosen reactions, while requiring that each such deletion preserves viability. Specifically, we chose a metabolic reaction at random and equiprobably among all reactions, deleted it, and used FBA to determine viability of the resulting metabolism. If the metabolism was viable, we accepted the deletion. Otherwise we randomly choose another reaction for deletion, and so on, until we found one whose deletion left the resulting metabolism viable. We also kept a count of the number of successive attempted deletions that resulted in a non-viable metabolism. This count was reset to zero if the deletion of a randomly chosen reaction was successful. Once that count reached 1000, that is 1000 successive attempts at reaction deletion abolished viability, we considered the metabolism a good candidate for a minimal metabolism. To confirm minimality, we deleted each reaction in this metabolism, and if every such deletion resulted in non-viability, we declared the metabolism to be minimal. The computer program required for generating viable potential metabolisms by random reaction deletion is available in Additional file 9.

### Identification of viability-preserving paths connecting viable genotypes G_1_ and G_2_ at arbitrary size n

To find out whether two genotypes *G*_1_ and *G*_2_ can be connected to one another through viability-preserving reaction swaps, we used the following heuristic approach. It does not rely on reaction swaps of arbitrary reactions, which we found to be too inefficient, but takes advantage of existing reactions in the two genotypes to accelerate the process. It defines a “walker” genotype *G*_1_ and alters it through multiple random steps (reaction swaps) that approach the other, “target” genotype *G*_2_. Before starting this walk, we established two lists of reactions *L*_*1*_ and *L*_*2*_. *L*_*1*_ contained all reactions in *G*_*1*_ that were not contained in *G*_*2*_. In this list we placed reactions non-essential in *G*_*1*_ first (and in random order), followed by reactions essential in *G*_*1*_ (also in random order). Conversely, *L*_*2*_ consisted of arbitrarily ordered essential reactions in *G*_*2*_, followed by arbitrarily ordered reactions nonessential in *G*_*2*_.

Each step in the random walk consisted of two parts, i.e., (i) adding to *G*_1_ a reaction from *L*_*2*_ (i.e., a reaction essential in *G*_2_), and (ii) deleting from *G*_1_ a reaction listed in *L*_*1*_. Subsequent steps used subsequent reactions in each list for addition and deletion.

As this walk through genotype space progressed, we continued adding reactions to *G*_1_ until all essential reactions from *G*_2_ in list *L*_*2*_ had been added to *G*_1_, and continued from there on to adding nonessential reactions from *L*_*2*_. During part (ii) of any given step, if none of the remaining reactions in the list could be deleted from walker *G*_1_ without losing viability, we reverted the last reaction addition, and chose instead a reaction at random from the universe as a candidate for addition. Before adding it, we ensured that the chosen reaction shared all of its substrates and products with other reactions in the random walker. If the product of the reaction was not shared with another reaction, we checked if it could be secreted by a transport reaction. A candidate reaction that did not fulfill both criteria would be disconnected from the rest of metabolism, could therefore not possibly contribute to viability [33], and we discarded it, choosing another candidate, and so on, until we had found one that fulfilled both criteria. We then determined if, after the addition of this reaction, some reaction in the list could be deleted from the random walker. If so, we accepted the resulting swap, otherwise we tried another addition, and so on, until we had found an acceptable swap.

The two parts of each step ensure, first, that essential reactions from the target are preferentially added to the walker, thus increasing the likelihood of adding “useful” reactions to *G*_1_, perhaps from one of several alternative metabolic pathways. Second, they reduce the chances of yielding an unviable genotype after a reaction deletion. However, the probability that the deletion of a reaction from walker *G*_1_ can render it unviable increases with the number of reaction swaps, because past steps may have rendered previously nonessential reactions essential. We therefore also needed to use FBA after each deletion to ensure that *G*_1_ retained viability after a reaction deletion.

We continued this guided random walk for as many swaps as needed to reach the target *G*_2,_ or until we had performed 5000 attempted swaps. In the latter case, we declared *G*_1_ and *G*_2_ disconnected. We note that this is no proof of disconnectedness, as some path may exist that this procedure cannot find. However, in practice, all our attempts to connect genotype pairs in this way were successful.

The computer program required for checking connectedness between a pair of metabolic genotypes using the above procedure is available as Additional file 9.

### Identification of a metabolism’s viable neighbors

Two metabolisms are adjacent or neighbors of each other with respect to a reaction swap if they differ by one such swap. If the focal metabolism contains *n* reactions, then there are *N-n* reactions that are not part of the focal metabolism, where *N* is the total number of reactions a metabolism could possibly have. One can thus obtain a neighbor of the focal metabolism by deleting one of its *n* reactions and simultaneously adding one of the *N*-*n* reaction from the universe of reactions. Any metabolism therefore has *n ×* (*N-n*) possible neighbors. To identify the viable neighbors of a minimal metabolism, we generated all possible *n* × (*N-n*) neighbors, and used FBA to determine their viability on glucose. (We also note that any minimal metabolism trivially has zero viable neighbors with respect to reaction deletion, and *N*-*n* viable neighbors with respect to reaction additions).

The computer program required for computing the viable neighbors of a metabolic genotype is available as Additional file 9.

We used MATLAB (Mathworks Inc.) for all numerical analysis. Genotype space visualization was generated using the script available at (http://www.oaslab.com/Drawing_funnels.html).

### Ethical considerations

This study is purely computational and does not use human or animal data. Ethical considerations do not apply.

## Competing interests

The authors declare that they have no competing interests.

## Authors’ contributions

AB, OCM and AW conceived the project, AB and SRH implemented code and performed computations, AB, OCM and AW wrote the manuscript. All authors read and approved the final manuscript.

## Supplementary Material

Additional file 1 **Representation of a genotype vector.** Any genotype encoding *n* reactions (*n* ≤ *N*) can be represented as a binary vector of length *N*, with *n* entries equal to one and all others equal to zero. The reactions that are present in the above hypothetical genotype are shown in black and the reactions that are absent are shown in grey. Click here for file

Additional file 2 Number of potential metabolisms viable on glucose with respect to metabolism size. Click here for file

Additional file 3 Number of potential metabolisms viable on all ten carbon sources as a function of metabolism size. Click here for file

Additional file 4 **An example of a minimal metabolism viable on glucose.** The figure shows an example of a minimal metabolism of size 23, which is also one of the smallest metabolisms viable on glucose. All 13 biomass precursors are framed with solid rectangles. Only important transport reactions and cofactors are shown. Enzymes catalyzing each of the reactions are shown in uppercase italic typeface. Abbreviations are spelled out in Additional file 2. Click here for file

Additional file 8 Number of metabolisms viable on all ten carbon sources as a function of metabolism size. Click here for file

Additional file 5 Supplementary results. Click here for file

Additional file 6 **Connectedness of genotype networks containing viable genotypes of *n* = 28, 29, and 30 reactions.** The figure shows the connectivity of genotype networks for reactions in central carbon metabolisms, as a function of size *n*. Each circle corresponds to a connected component, and the number in each circle corresponds to the number of genotypes in this component. The components at size 28 were obtained by full enumeration, but for larger sizes such an approach is not feasible. Instead one has to use a form of recursive evaluation that we illustrate here for two larger sizes. Panel **(A)** shows the two disconnected components in the network corresponding to size 28, one containing 434234 genotypes, and the other component containing just 4 genotypes. The addition of one reaction to these 4 genotypes results in 88 genotypes of size 29, which must be connected (see main text). **(B)** Aside from these 88 connected genotypes, there are also 28 minimal genotypes of size 29 (red circles). We verified computationally that both groups of genotypes (88 and 29) were connected using breadth-first search and found that they form a single component of 116 genotypes. We were also able to demonstrate that this component is connected to the 1773853 connected genotypes that are parents of the large component at size *n* = 28 (panel A). The two thus form a connected genotype network of 1773969 metabolisms of size 29. Panel **(C)** shows that adding one reaction to these genotypes results in 5900563 connected genotypes at size 30. In addition, 15 new minimal metabolisms (red circles) come into being at size 30. We found that they were connected to the remaining 5900563 genotypes, thus forming a single connected network comprising 5900578 genotypes. Click here for file

Additional file 7 **An example of essential pathways that result in fragmentation of genotype space.** The figure shows the two essential pathways (green and blue) in a pair of genotypes belonging to the two disconnected components in the genotype network of potential metabolisms with *n* = 25 reactions (Figure 3C). The reactions in green, catalyzed by enzymes PFK, FBAl and TPI are essential in all genotypes belonging to the largest component (subgraphs *A”* and *B”*) in Figure 3C, while the reactions in blue are essential to all four genotypes in component *C* in Figure 3C. The 13 biomass precursors are surrounded by black rectangles. Only important transport reactions and cofactors have been shown. Enzymes catalyzing each of the reactions are shown in uppercase italic typeface. Information on abbreviations is provided in Additional file 2. Click here for file

Additional file 9 The essential computer programs used in this analysis. Click here for file
