# Supplementary material for: Historical contingency and the gradual evolution of metabolic properties in central carbon and genome-scale metabolisms
Source: BMC Syst Biol. 2014 Apr 23;8:48. doi: 10.1186/1752-0509-8-48 (PMC4022055; doi:10.1186/1752-0509-8-48)
Supplement: Additional file 1 — Representation of a genotype vector. Any genotype encoding n reactions (n ≤ N) can be represented as a binary vector of length N, with n entries equal to one and all others equal to zero. The reactions that are present in the above hypothetical genotype are shown in black and the reactions that are absent are shown in grey. [file 1752-0509-8-48-S1.pdf]

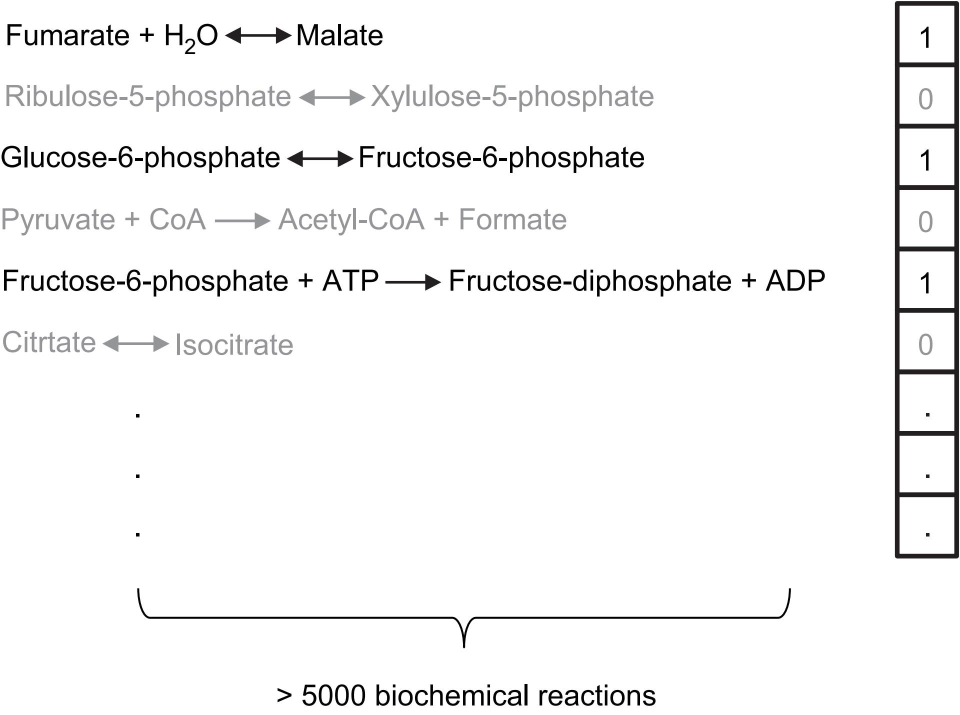

Fumarate + H_2O ⟷ Malate
Ribulose-5-phosphate ⟷ Xylulose-5-phosphate
Glucose-6-phosphate ⟷ Fructose-6-phosphate
Pyruvate + CoA ⟶ Acetyl-CoA + Formate
Fructose-6-phosphate + ATP ⟶ Fructose-diphosphate + ADP
Citrtate ⟷ Isocitrate
1
0
1
0
1
0
> 5000 biochemical reactions
