# Supplementary figures and images for: Historical contingency and the gradual evolution of metabolic properties in central carbon and genome-scale metabolisms
Source: BMC Syst Biol. 2014 Apr 23;8:48. doi: 10.1186/1752-0509-8-48 (PMC4022055; doi:10.1186/1752-0509-8-48)

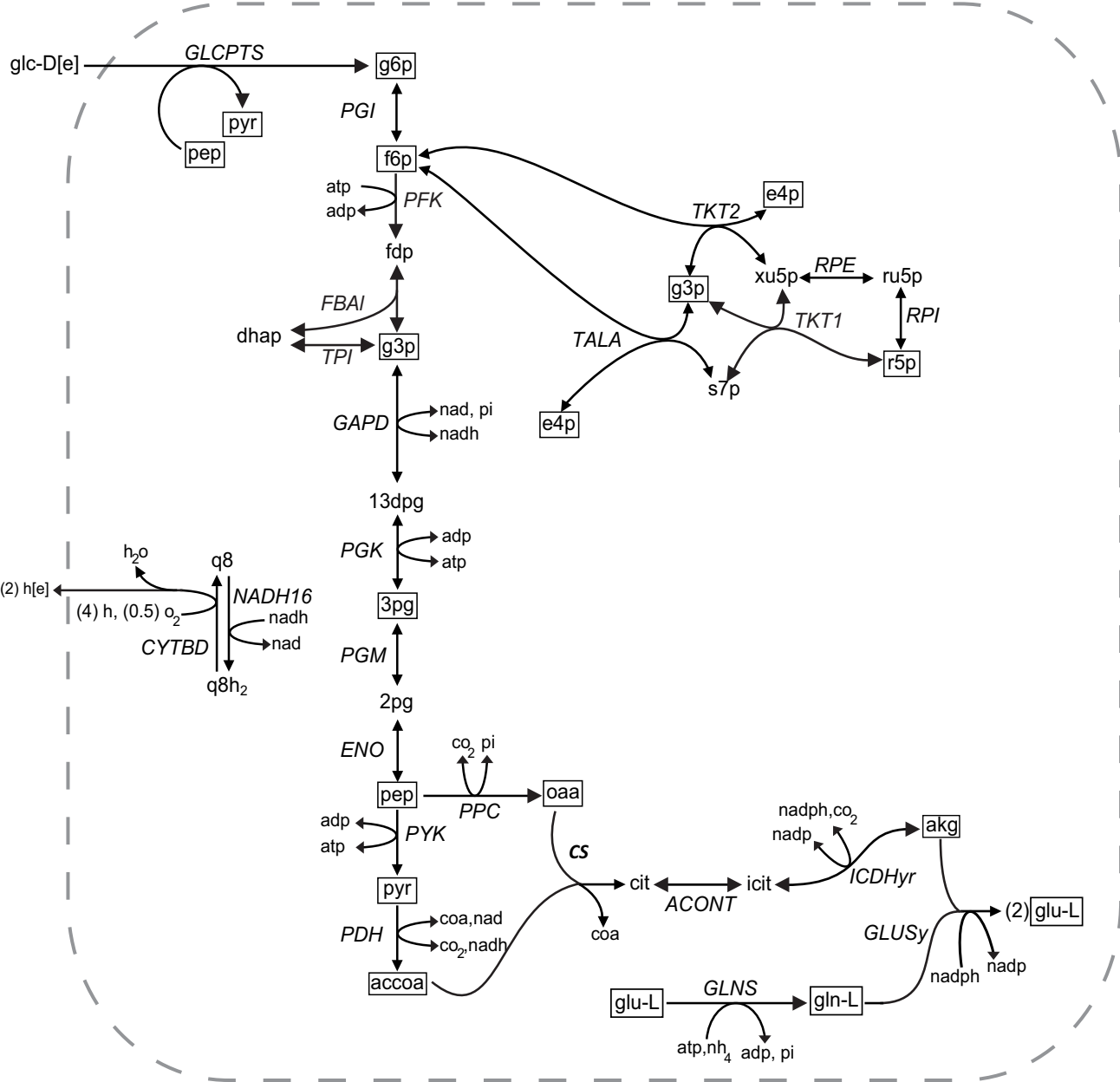

Supplement: Additional file 4 — An example of a minimal metabolism viable on glucose. The figure shows an example of a minimal metabolism of size 23, which is also one of the smallest metabolisms viable on glucose. All 13 biomass precursors are framed with solid rectangles. Only important transport reactions and cofactors are shown. Enzymes catalyzing each of the reactions are shown in uppercase italic typeface. Abbreviations are spelled out in Additional file 2. [file 1752-0509-8-48-S4.pdf]

**A** $n = 28$ 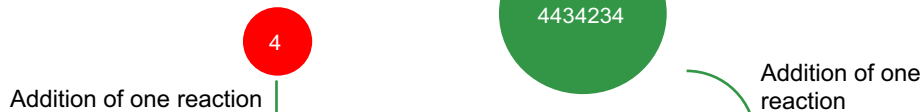**B** $n = 29$ 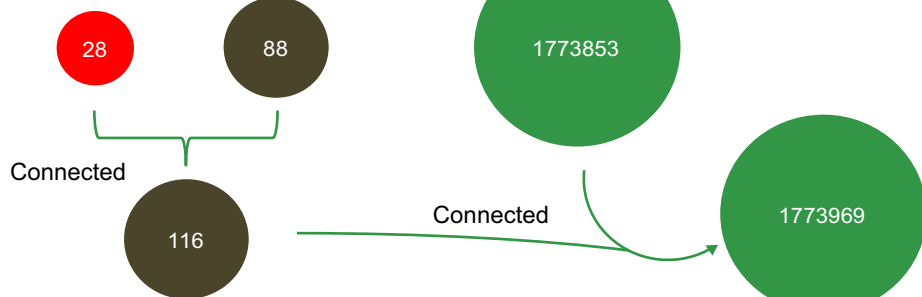**C** $n = 30$ 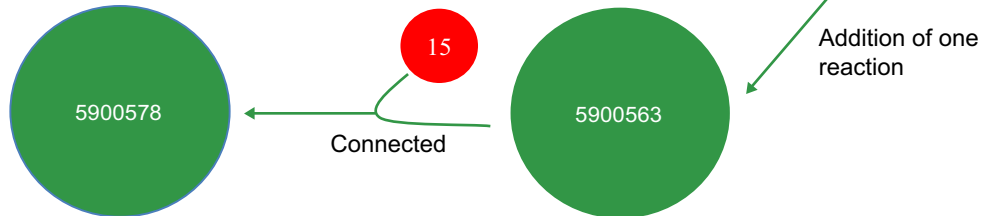

Supplement: Additional file 6 — Connectedness of genotype networks containing viable genotypes of n = 28, 29, and 30 reactions. The figure shows the connectivity of genotype networks for reactions in central carbon metabolisms, as a function of size n. Each circle corresponds to a connected component, and the number in each circle corresponds to the number of genotypes in this component. The components at size 28 were obtained by full enumeration, but for larger sizes such an approach is not feasible. Instead one has to use a form of recursive evaluation that we illustrate here for two larger sizes. Panel (A) shows the two disconnected components in the network corresponding to size 28, one containing 434234 genotypes, and the other component containing just 4 genotypes. The addition of one reaction to these 4 genotypes results in 88 genotypes of size 29, which must be connected (see main text). (B) Aside from these 88 connected genotypes, there are also 28 minimal genotypes of size 29 (red circles). We verified computationally that both groups of genotypes (88 and 29) were connected using breadth-first search and found that they form a single component of 116 genotypes. We were also able to demonstrate that this component is connected to the 1773853 connected genotypes that are parents of the large component at size n = 28 (panel A). The two thus form a connected genotype network of 1773969 metabolisms of size 29. Panel (C) shows that adding one reaction to these genotypes results in 5900563 connected genotypes at size 30. In addition, 15 new minimal metabolisms (red circles) come into being at size 30. We found that they were connected to the remaining 5900563 genotypes, thus forming a single connected network comprising 5900578 genotypes. [file 1752-0509-8-48-S6.pdf]

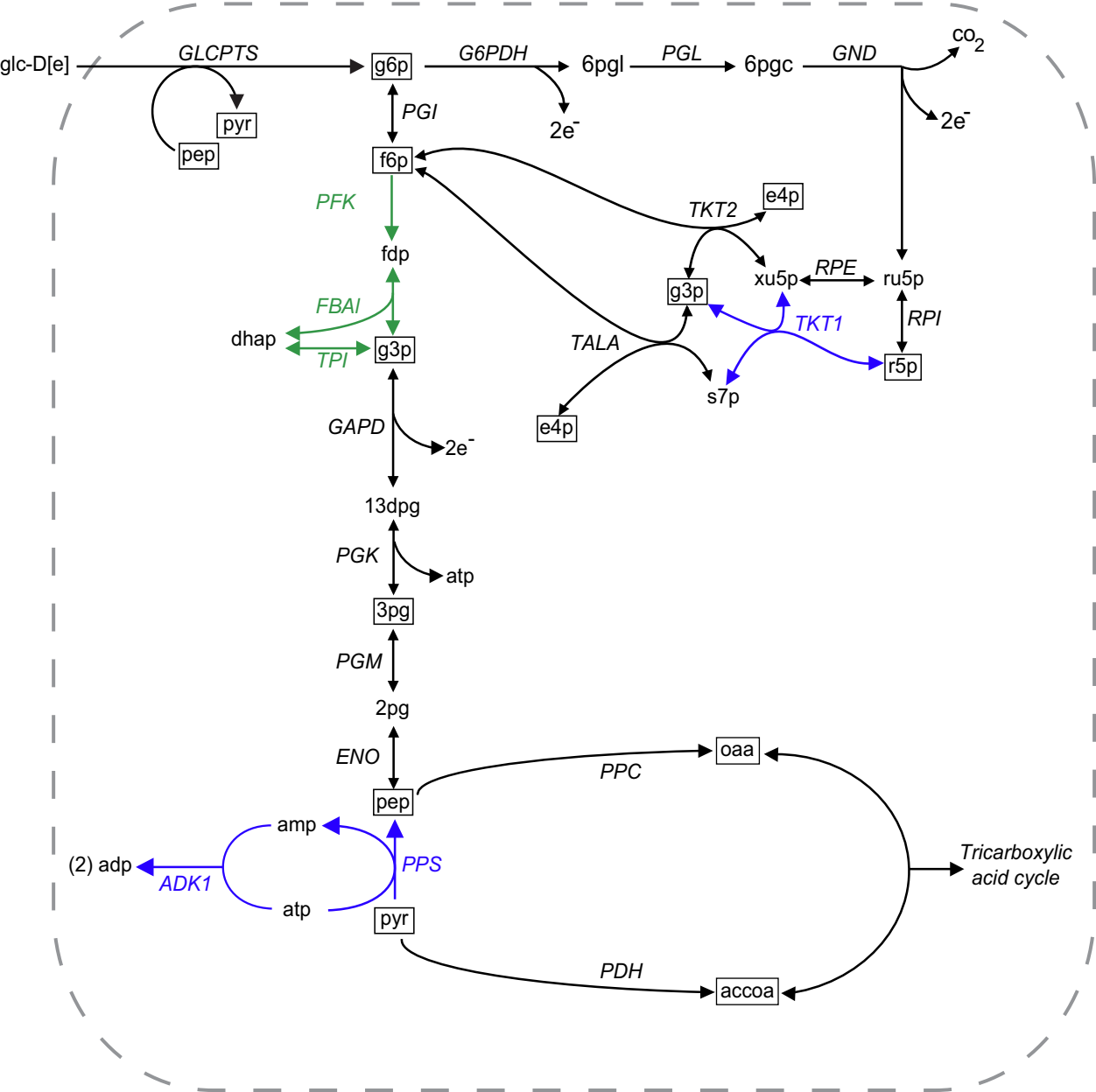

Supplement: Additional file 7 — An example of essential pathways that result in fragmentation of genotype space. The figure shows the two essential pathways (green and blue) in a pair of genotypes belonging to the two disconnected components in the genotype network of potential metabolisms with n = 25 reactions (Figure 3C). The reactions in green, catalyzed by enzymes PFK, FBAl and TPI are essential in all genotypes belonging to the largest component (subgraphs A” and B”) in Figure 3C, while the reactions in blue are essential to all four genotypes in component C in Figure 3C. The 13 biomass precursors are surrounded by black rectangles. Only important transport reactions and cofactors have been shown. Enzymes catalyzing each of the reactions are shown in uppercase italic typeface. Information on abbreviations is provided in Additional file 2. [file 1752-0509-8-48-S7.pdf]
