# Supplementary material for: Historical contingency and the gradual evolution of metabolic properties in central carbon and genome-scale metabolisms
Source: BMC Syst Biol. 2014 Apr 23;8:48. doi: 10.1186/1752-0509-8-48 (PMC4022055; doi:10.1186/1752-0509-8-48)
Supplement: Additional file 5 — Supplementary results. [file 1752-0509-8-48-S5.pdf]

The connectivity of central carbon metabolisms with $n \geq 29$ reactions

We used the software `igraph` [1] to determine connectedness of genotype networks for potential metabolisms of size 23-28 and 46-50. We found that these genotype networks are mostly connected, as discussed in the main text. Unfortunately, that approach proved computationally too demanding for exploring the connectedness of potential metabolisms of greater sizes. We thus used another method to assess whether genotype networks of potential metabolisms at intermediate sizes are connected. At the heart of our method is the observation that if some subgraph A of a genotype network containing genotypes of size n is connected, then the genotypes $G(n+1)$ obtained from A by a single reaction addition also form a connected set. To illustrate our approach for small values of n, we start with the two components in the genotype network of size n=28. Panel A in Additional file 6 displays the two components in the genotype network corresponding to size 28, one containing 434234 genotypes, and the other containing just 4 genotypes. By adding one reaction to each of these genotypes, we generate potential metabolisms of size 29. The two corresponding sets have 1773853 and 88 genotypes respectively (Panel B in Additional file 6), in addition to the remainder of 28 viable metabolisms of size 29. These 28 potential metabolisms are minimal in nature. Because these genotypes are minimal, they cannot belong to the two sets that were generated by the reaction addition process. Taking these new genotypes into account, one arrives at the total number of viable genotypes in $V(29)$ (Panel B in Additional file 6 and Table 1).

The arguments in the main text (section "Determining connectedness of genotype networks") show why each set obtained by reaction addition must be connected, but they do not tell us whether these two components are connected to one another, nor do they not inform us how the 28 minimal metabolisms connect to them. To determine their connectedness, we first checked if the 28 minimal metabolisms form a connected set using `igraph` [1]. We repeated the procedure and found that these 28 minimal metabolisms and the set of 88 potential metabolisms are also connected, yielding a larger component of size 116 (Panel B in Additional file 6). We next checked if any one of these 116 genotypes is separated by a reaction swap (constituting a neighbor) from another genotype in a set of 10000 sampled genotypes from the larger component of 1773853 genotypes. We found many pairs that were

neighbors, demonstrating that metabolisms of size 29 form one fully connected genotype network.

The analysis can be extended to larger sizes. By our argument in the main text, all genotypes of size 30 that derive from genotypes of size 29 by addition of one reaction form a connected set. In addition to these 5900563 genotypes (Panel C in Additional file 6), potential metabolisms at size 30 also contain 15 new minimal metabolisms (Panel C in Additional file 6 and Table 1). We examined them in the same way as described above, and found them all to be connected to the much larger component. In other words, all viable metabolisms with n = 30 reactions form a single connected component. At each step thereafter in this recursive reasoning to go from n to n+1, it is enough to determine all minimal metabolisms at size n+1 and to verify that they are neighbors of genotypes obtained by one reaction addition to viable genotypes at size n.

Essential pathways cause genotype network fragmentation

We here discuss another example that illustrates why pathway essentiality can lead to genotype network fragmentation. This one involves genotypes from the large connected component of size 25 (subgraphs *A''* and *B''* in Figure 3C), on the one hand, and component *C* (blue in Figure 3C), on the other. Again, we first calculated the superessentiality indices of all reactions for these components, and examined which reactions differ in their superessentiality index between the two components. We find that reactions catalyzed by phosphofructokinase (PFK), fructose-biphosphate aldolase (FBAl), and triose phosphate isomerase (TPI) are essential in all genotypes in the large component (Additional File 7, in green). Conversely, reactions catalyzed by phophoenolpyruvate synthase (PPS) and adenylate kinase (ADK1) (blue, Additional File 7) are essential in all four genotypes belonging to component *C* (Figure 3C). For the purpose of illustration, Additional File 7 indicates in the form of one figure, the differences between a pair of genotypes (G_1, G_2), where G_1 belongs to the large component in Figure 3C (subgraphs *A''* and *B''*, genotype includes reactions in green, but does not contain reactions in blue – see Additional File 7) and the other genotype G_2 belongs to component *C* in Figure 3C (genotype includes reactions in blue, but does not contain reactions in green – see Additional File 7). Reactions in black are encoded by both genotypes G_1 and G_2. We chose these specific genotypes G_1 and G_2 because they are separated by three reaction swaps, which is also the minimal distance between a pair of

metabolisms of size 25 belonging to different components. Unlike the example discussed in the main text, where the essential pathways formed alternative routes (Figure 4), these two essential pathways do not form alternative routes towards synthesizing the same molecules.

In addition to reactions catalyzed by PPS and ADK1, genotype G_1 does not contain the reaction catalyzed by TKT1, and is thus unable to synthesize sedeheptulose-7-phosphate (s7p). Though the reaction catalyzed by TALA is present in G_1 (as well as in G_2), it is nonfunctional in G_1 as s7p is one of its substrates. This also means that ribose-5-phosphate (r5p) and erythrose-4-phosphate (e4p) are synthesized through the pathway catalyzed by reactions G6PDH, PGL and GND (Additional File 7) as described in the example in the main text.

In genotype G_2, the absence of reactions PFK, FBAl and TPI (Additional File 7) forces all carbon that enters central carbon metabolism in the form of glucose-6-phosphate (g6p) towards the pentose phosphate pathway via the reaction catalyzed by glucose-6-phosphate dehydrogenase (G6PDH). Flux balance analysis shows that this results in most of the carbon being secreted as carbon dioxide (reaction GND (Additional File 7), while a minority of it can enter the lower part of glycolysis as g3p and is converted further through GAPD, PGK, PGM and ENO into phosphoenolpyruvate (pep, Additional File 7). Secretion of a majority of carbon in the form of carbon dioxide results in the flux in the lower half of glycolysis to be smaller than the flux at which glucose-6-phosphate is generated in genotype G_2. However, pep needs to be generated at the same rate as glucose is imported through the glucose phosphotransferase system (GLCPTS) to maintain steady state. Because the flux in the lower half of glycolysis is smaller than the upper half, there is a flux deficit in the rate at which phosphoenolpyruvate is synthesized.

This flux deficit is rectified by phosphoenolpyruvate synthase (PPS). PPS is thus necessary in G_2 for the biosynthesis of the required amount of phosphoenolpyruvate (pep). While PPS fulfills these demands, it also generates adenosine monophosphate (AMP) as a by-product. To ensure mass balance, this AMP needs to be utilized, which can be accomplished by adenylate kinase (ADK1) (Additional File 7). The reaction that ADK1 catalyzes is, in fact, the only reaction in our central carbon metabolism model that utilizes AMP. Thus, the requirement of phosphoenolpyruvate renders reactions PPS and ADK1 essential. Furthermore, because reactions catalyzed by PFK, FBAl and TPI are absent in G_2 reactions catalyzed by G6PDH, PGL, GND, TKT1 and TALA are all essential in this genotype.

In light of these observations, one also might ask why genotype networks of potential metabolisms with 25 and 26 reactions are disconnected (Figure 2A and Figure 3C), while genotype networks of parent metabolisms with 27 reactions are connected (Figure 2A). As mentioned above, genotypes G_1 and G_2 are of size 25 and separated by three reaction swaps. Reactions catalyzed by PFK, FBAl and TPI are present in genotype G_1 and not in G_2, while reactions PPS, ADK1 and TKT1 are present in genotype G_2, but not in G_1. To generate parent metabolisms of size 26 from G_1 and G_2, we could add PPS to G_1 resulting in G_1', while adding PFK to G_2 would result in G_2'. Genotypes G_1' and G_2' would now be separated by two reaction swaps. Repeating this process by addition of ADK1 to G_1' and FBAl to G_2' would result in genotypes G_1'' and G_2'' of size 27, which are separated by one reaction swap and thus connected. That is, G_1'' could now be converted to G_2'' by addition of TKT1 and removal of TPI (Additional File 7).

References

1. Csardi G, Nepusz T: **The igraph software package for complex network research**. *InterJournal Complex Systems* 2006, **1695**:1695.
