# Supplementary material for: Historical contingency and the gradual evolution of metabolic properties in central carbon and genome-scale metabolisms
Source: BMC Syst Biol. 2014 Apr 23;8:48. doi: 10.1186/1752-0509-8-48 (PMC4022055; doi:10.1186/1752-0509-8-48)
Supplement: Additional file 9 — The essential computer programs used in this analysis. [file 1752-0509-8-48-S9.zip › Additional File 9/README.doc]

**README**

**Instructions for installing and using essential computer programs utilized in this manuscript**

Enclosed with this document, there are the following files.

**Programs:**

fba-solve : computes rate of biomass synthesis.

find-essential : identifies essential reactions

find-minimalmetab : computes a minimal metabolism

metab-walktoward : finds a path connecting two metabolisms

find-neighbormetabs : counts the number of neighboring metabolisms

**Input files:**

iAF1260.net : the *E. coli* model by Feist *et. al.* [1]. Contains 1397 cytoplasmic and 682 transport reactions.

minimalmetab.net : a minimal metabolism

net600-1.net : a metabolism of containing 600 reactions

net600-2.net : another metabolism of containing 600 reactions

env_glucose.flx : environment file containing glucose as the sole carbon source

The programs included are used and tested on Ubuntu Version 10, a flavor of the Linux operating system. Before using these programs, one needs to install the following dependencies. Below, we list the standard method for installing them, which requires root access. In case you install these programs in a directory different from their default location, you may need to export the relevant path, such as LD_LIBRARY_PATH, so that the FBA programs can find the packages they depend on during run time.

sudo apt-get install g++

sudo apt-get install fort77

sudo apt-get install automake

sudo apt-get install libgmp3-dev

sudo apt-get install libreadline5-dev

sudo apt-get install libpthread-stubs0-dev

sudo apt-get install libgsl0ldbl

sudo apt-get install libgsl0-dev

sudo apt-get install libz-dev

sudo apt-get install subversion

One also needs to install a basic linear programming solver, such as CLP for carrying out flux balance analysis (FBA). The latest stable version of CLP is 1.9. Here are installation instructions for this version:

svn co https://projects.coin-or.org/svn/Clp/stable/1.9 coin-Clp

cd coin-Clp

sudo ./configure -C --prefix=/usr/local/

sudo make

sudo make test (# All tests should pass)

sudo make install

sudo ldconfig

cd ..

Note that these instructions install CLP in the system directory /usr/local. If you install elsewhere, do not forget to change the LD_LIBRARY_PATH accordingly. Once you have successfully installed all these dependencies, we can proceed with the usage of the FBA-related programs.

**Structure of input files**

The metabolism files we use here have a nonstandard representation which is briefly explained below. Each line represents a reaction, such as

ECOLI_2,PPPGO: (1.5) C00007 + C01079 --> (3) C00001 + C02191

where ECOLI_2 is our unique identifier for the reaction and PPPGO is the identifier from the published model of *E. coli* metabolism by Feist *et. Al* [1]. These words are followed by the reaction equation, which represent metabolites in KEGG nomenclature [2,3]. Metabolites not found in KEGG (see Methods in the main text for further details) have the same nomenclature as in [1]. Suffix [e] denotes a metabolite in the extracellular compartment, suffix [p] denotes a metabolite in the periplasm, and the absence of any suffix denotes the cytoplasm. The arrow --> denotes an irreversible reaction and <==> denotes a reversible reaction. FBA also needs an environment file, such as the file called env_glucose.flx. The environment file contains information about the nutrients and their maximum uptake rates. For example,

C00031[e] = -10

C00007[e] = -20

C00076[e] = -1000

Here, the first line denotes the uptake rate (10 mmol/gram dry weight/hour) of extracellular glucose. (C00031 is glucose, C00007 is oxygen (O_2_) and C00076 is calcium (Ca_2_^+^)). Note that all nutrients excluding glucose and oxygen are supplied in essentially unlimited amounts (1000 mmol/gDW/hr).

**Flux balance analysis to compute rate of synthesis of biomass precursors**

From the directory which contains the files mentioned above, at the command prompt, type the following:

./fba-solve iAF1260.net env_glucose.flx

you should see the following output

<11esolver_clp>

\# Metabolism: iAF1260.net

\# Biomass synthesis rate : env_glucose.flx : 0.962955

The first line denotes the solver you are using, the second line denotes which metabolism is being used and the third line contains the name of the environment file used to compute FBA, followed by the rate of synthesis of biomass precursors (the biomass growth flux) in mmol/gDW/hr.

**Identification of essential reactions**

For the identification of essential reactions, type the following at the command prompt:

./find-essential iAF1260.net env_glucose.flx

you should see output that begins with the following lines

```
	<11esolver_clp>
	# Metabolism: iAF1260.net
	# Biomass synthesis rate : env_glucose.flx : 0.962955
	# Essential reactions:
	ECOLI_3
	ECOLI_5
	ECOLI_11
...
```

Each line beyond the fourth line contains the name of a reaction essential to synthesize biomass precursors. At the end, the program also prints the total number of essential reactions found. For the *E. coli* metabolism, it is

\# Number of nonessential reactions: 240

**Finding minimal metabolisms**

To find minimal metabolisms, one needs to provide a larger metabolism that the minimal metabolism would be a subset of. Results may vary depending on this larger metabolism. In the following example, the *E. coli* metabolism iAF1260.net [1] is provided. The syntax for computing a minimal metabolism is

./find-minimalmetab [--netfile minimalmetab.net] iAF1260.net	env_glucose.flx

wherein the argument --netfile is followed by the name of the file that will contain the minimal metabolism generated by the program. You should see output similar to that described below

```
	<11esolver_clp>
	# Generating minimal network from: iAF1260.net
	ECOLI_723,DUTPDP: C00460 + C00001 --> C00365 + C00080 + C00013 : 	1396
	ECOLI_717,NTD5: C00364 + C00001 --> C00009 + C00214 : 1395
...
```

wherein the each line consists of the randomly chosen reaction that was deleted, followed by the number of cytoplasmic reactions in the metabolism, separated by the delimiter :. By default, the minimal metabolism will be saved as minimalmetab.net. One can check the biomass synthesis rate of the minimal metabolism by using the fba-solve program described above.

**Walk from genotype *G_1_* to *G_2_* to test connectedness**

This programs allows one to test if two non-minimal metabolisms can be connected through a series of reaction swaps. The following example uses the metabolisms in files net600-1.net and net600-2.net for this purpose. Both contain 600 cytoplasmic reactions (as well as 682 transport reactions) and are a subset of the *E. coli* metabolism. To test connectedness, type the following at the command prompt:

./metab-walktoward iAF1260.net net600-1.net net600-2.net	env_glucose.flx

You should see the following output:

```
	<11esolver_clp>
	# Reference metabolism: net600-1.net
	# Finding essential reactions in ref. metabolism ...
	# Number of essential reactions in ref. metabolism: 342

	# Walk-with metabolism: net600-2.net
	# Finding essential reactions in the walker metabolism ...
	# Number of essential reactions in the walker metabolism: 321

	# Number of common reactions: 1045

	# Hamming distance between net1 and net2 for cytoplasmic 	reactions: 0.395
	# Distance (in reactions) between net1 and net2 for cytoplasmic 	reactions: 237
	# Maximum iterations allowed: 2370

	# Starting walk from net600-2.net to net600-1.net
	#+ ECOLI_1054
	#- ECOLI_549
	Distance between metabolisms is now: 0.393333, iteration: 2
	...
```

The line #+ ECOLI_1054 means that reaction ECOLI_1054 has been added to the “waker” metabolism, and #- ECOLI_549 means that reaction ECOLI_549 has been deleted from it. If the walk succeeds, one should see the following output:

End of walk : success, found a path from net600-2.net to	net600-1.net

**Counting neighboring metabolisms**

To compute the number of neighboring metabolisms, that is, metabolisms separated by one swap from the focal minimal metabolism, type the following:

./find-neighbormetabs iAF1260.net minimalmetab.net	env_glucose.flx

The minimal metabolism minimalmetab.net in this example is generated from the *E. coli* metabolism iAF1260.net. You should see the following output:

\# Superset metabolism: iAF1260.net

\# Computing neighbors for metabolism: minimalmetab.net

<11esolver_clp>

\# Reaction being replaced : Number of neighbors : Cumulative	number of neighbors

ECOLI_3 : 0 : 0

ECOLI_5 : 0 : 0

ECOLI_11 : 0 : 0

ECOLI_19 : 0 : 0

ECOLI_32 : 0 : 0

ECOLI_33 : 0 : 0

ECOLI_38 : 1 : 1

ECOLI_39 : 0 : 1

Each line of the output contains, from left to right, the name of the reaction being deleted. The second column denotes the number of viable metabolisms found in the following manner. For each deletion, the program adds one reaction and checks for viability of that metabolism (see Methods). If the metabolism is viable, it adds to the count of viable metabolisms found. It then deletes the added reaction and adds another and repeats the process for all reactions. The third column denotes the running total of all viable metabolisms found until that step, that is, the cumulative number of neighbors. The pool of reactions used to compute the neighbors is taken from the superset metabolism, in this example iAF1260.net.

**Note**

For any of the above programs, the usage syntax can be checked by just typing the name of the program. For example, to check the usage syntax of the program fba-solve, one can type:

./fba-solve

One should see the following output:

E! [] int emain()(): !error!: Usage: ./fba_solve	<metabolism.net> <environment.flx>

**Bibliography**

1. Feist AM, Henry CS, Reed JL, Krummenacker M, Joyce AR, Karp PD, Broadbelt LJ, Hatzimanikatis V, Palsson BØ: **A genome-scale metabolic reconstruction for Escherichia coli K-12 MG1655 that accounts for 1260 ORFs and thermodynamic information.** *Molecular systems biology* 2007, **3**:121.

2. Goto S, Okuno Y, Hattori M, Nishioka T, Kanehisa M: **LIGAND: database of chemical compounds and reactions in biological pathways.** *Nucleic acids research* 2002, **30**:402–4.

3. Goto S, Nishioka T, Kanehisa M: **LIGAND: chemical database of enzyme reactions.** *Nucleic acids research* 2000, **28**:380–2.
